# Contrasting modes of intron evolution in an evolutionarily distinct eukaryotic lineage revealed by comparative genomics of ciliated protists

**DOI:** 10.1093/molbev/msag252

**Published:** 2026-09-30

**Authors:** Zhaorui Zhou, Ruitao Gong, Yong Chi, Yurui Wang, Zhiqiang Ye, Ying Yan

**Affiliations:** Key Laboratory of Evolution and Marine Biodiversity (Ministry of Education), and Institute of Evolution and Marine Biodiversity, Ocean University of China, Qingdao 266003, China; Key Laboratory of Evolution and Marine Biodiversity (Ministry of Education), and Institute of Evolution and Marine Biodiversity, Ocean University of China, Qingdao 266003, China; Marine College, Shandong University, Weihai 264209, China; Laboratory of Biodiversity and Evolution of Protozoa in Wetland, College of Life Sciences, Shaanxi Normal University, Xi’an 710119, China; Key Laboratory of Pesticide and Chemical Biology of Ministry of Education, School of Life Sciences, Central China Normal University, Wuhan 430079, China; Key Laboratory of Evolution and Marine Biodiversity (Ministry of Education), and Institute of Evolution and Marine Biodiversity, Ocean University of China, Qingdao 266003, China

**Keywords:** intron evolution, intron phase, ciliates, spliceosome, whole-genome duplication

## Abstract

Introns are widespread in eukaryotic genomes and play important roles in gene regulation, making intron evolution a long-standing focus of genome research. However, the patterns and mechanisms of intron evolution in evolutionarily ancient unicellular eukaryotes remain poorly understood. Here, we investigate intron evolution in ciliates, a morphologically and genetically diverse unicellular lineage, using a newly sequenced high-quality genome and comparative genomic data from derived and deep-branching lineages. Our analyses reveal that: (i) intron evolution in ciliates has been dominated by loss. While derived lineages experienced limited loss, the common ancestor of deep-branching groups, represented by heterotrich ciliates, underwent massive intron loss with almost no detectable gain. (ii) Unlike derived ciliates, which display relatively uniform distributions of 3*n*, 3*n* + 1, and 3*n* + 2 introns or a low proportion of 3*n* introns, deep-branching ciliates show a striking dominance of tiny 3*n* introns. These are preferentially retained when generating in-frame premature termination codons (PTCs), likely allowing aberrant transcripts to be eliminated via nonsense-mediated mRNA decay, while introns incapable of forming PTCs tend to avoid insertion within protein domains, suggesting a strategy to reduce the metabolic cost of erroneous splicing. (iii) Spliceosome analyses reveal conserved core catalytic components but lineage-specific variation in regulatory factors, suggesting differences in splicing regulation among ciliate lineages. Furthermore, the newly assembled 48.5 Mb *Blepharisma* genome encodes 27,571 protein-coding genes, and comparative analyses identified two rounds of whole-genome duplication in *Blepharisma*, one shared with *Stentor*. These findings provide new insights into intron evolution in unicellular eukaryotes and highlight ciliates as a powerful model for studying unconventional intron evolutionary strategies.

## Introduction

The origin of introns and the evolutionary forces acting on them have long been central questions in evolutionary biology. As a defining feature of eukaryotic genomes, introns have attracted significant attention since their discovery in the 1970s, giving rise to extensive theoretical and empirical research (Gilbert 1978; Rogozin et al. 2003, 2012; Jo and Choi 2015; Girardini et al. 2023; Xu et al. 2023). Current consensus suggests that modern eukaryotic spliceosomal introns likely originated from ancestral group II self-splicing introns, which entered early eukaryotic genomes through endosymbiotic gene transfer and subsequently evolved into the spliceosomal intron system (Chalamcharla et al. 2010; Rogozin et al. 2012). This transition was accompanied by complex adaptive processes, including the optimization of splicing signals and the co-evolution of the spliceosomal machinery (Sales-Lee et al. 2021).

Phylogenetic reconstructions generally support the hypothesis that the Last Eukaryotic Common Ancestor harbored an intron-rich genome (Carmel et al. 2007; Csűrös et al. 2011; Grau-Bové et al. 2017). However, this ancestral condition contrasts sharply with the intron-poor genomes observed in many extant eukaryotic lineages, such as microsporidia (Lim et al. 2021) and some apicomplexans with small-genomes (Yeoh et al. 2019), highlighting the complex and dynamic nature of intron evolution. The evolutionary trajectories of intron-exon structures are far from a unidirectional process of genome simplification. Instead, they display remarkable lineage-specific diversification: some groups, such as mammals (Grau-Bové et al. 2017) and dinoflagellates (Roy et al. 2023), have retained or even expanded their intron content, whereas others, such as certain fungi, have experienced extensive intron loss (Lim et al. 2021). Early comparative studies across 99 eukaryotic genomes revealed the extreme variability in intron densities among lineages (Csűrös et al. 2011), a pattern further corroborated by recent focused investigations in nematodes (Ma et al. 2022), fungi (Lim et al. 2021), and other clades (Li et al. 2014; Cissé et al. 2021; Cicconardi et al. 2023).

Several mechanisms have been proposed to explain the dynamic turnover of introns. Intron loss is commonly attributed to homologous recombination between genomic loci and reverse-transcribed mRNA, or to genomic deletions (Niu et al. 2005; Cohen et al. 2012; Odom and Herrin 2013). Intron gains, on the other hand, may arise through a variety of mechanisms, including intronization of exonic sequences, tandem genomic duplication, intron gain during DNA double-strand break repair, intron transfer, and insertion of transposable elements such as introners (Yenerall et al. 2011; Yenerall and Zhou 2012; Gozashti et al. 2022; Roy et al. 2023; Gozashti et al. 2025).

Despite progress in understanding intron evolution in several eukaryotic groups, existing studies often rely on comparisons across distantly related taxa, constraining resolution and inference. A species-rich, monophyletic lineage with pronounced intron diversity is therefore essential for dissecting intron evolutionary patterns and mechanisms. As one of the most diverse and evolutionarily successful lineages of unicellular eukaryotes that emerged approximately one billion years ago, ciliates offer a unique window into intron evolution within a complex unicellular system (Gao et al. 2025; Wang et al. 2026). These organisms are renowned for their extraordinary morphological diversity and possess unusually dynamic genome architectures, including large-scale genome rearrangements, distinct epigenetic landscapes, and highly fragmented chromosomes (Lyu et al. 2024, 2025; Jiang et al. 2025; Wang et al. 2025; Ye et al. 2025). Despite these intriguing genomic features, the structure and functional significance of introns in ciliates remain poorly understood.

Emerging evidence indicates substantial lineage-specific differences in intron abundance across ciliates. Of particular interest, the first macronuclear genome assembly of a heterotrich ciliate revealed a highly unusual intron landscape: a low overall intron density coupled with the dominance of extremely short introns, primarily 15 base pairs (bp) in length (Slabodnick et al. 2017). This observation raises several fundamental questions: How diverse are intron structures across ciliates? What drives the substantial variability in intron density among different ciliate lineages? What are the specific advantages of adopting ultra-short introns as the primary form in heterotrich ciliates?

To address these questions, we newly sequenced and assembled the macronuclear genome of *Blepharisma undulans*, a representative heterotrich ciliate. Comparative genomic analyses revealed, for the first time, two whole-genome duplication events in the genus *Blepharisma*, one of which is shared with *Stentor coeruleus*. Different duplicate types exhibit marked divergence in selection pressure and contribution to gene family expansion. Together with publicly available ciliate genome data, we systematically characterize the intron diversity and evolutionary dynamics within the phylum Ciliophora. We focused on the intron distribution, phase composition, and sequence features, and examined their potential roles in maintaining gene expression robustness. In addition, on the basis of rigorously defined criteria for sequence similarity assessment, we identified and performed detailed analyses of spliceosomal proteins and snRNAs across ciliates, enabling an evolutionary evaluation of splicing mechanisms in this lineage. We aim to provide new perspectives on intron evolution and offer an important model for understanding the interplay between genome architecture, translational accuracy, and molecular-level error control.

## Results

### Genomic signatures in *B. undulans*

We employed PacBio high-fidelity (HiFi) long-read sequencing to generate high-quality contigs for *B. undulans*. A summary of statistics for macronuclear genome assembly and total raw reads is shown in Table S1. Based on k-mer analysis with about 474× coverage short reads, the size of the macronuclear genome was estimated to be 42.2 Mb (Fig. S1). The final assembled genome size is 48.5 Mb with GC content of 32.5%. The contig N50 length is 457,113 bp (Fig. 1a). A prominent feature of the *Blepharisma* macronuclear genome is its extensive fragmentation, characterized by the widespread distribution of telomere addition sites across individual contigs, resulting in a unique genome architecture composed of minichromosomes whose sizes range between typical long chromosomes and nanochromosomes (Singh et al. 2023). This is reflected by the broad distribution of telomeric reads throughout the *B. undulans* macronuclear genome. Using a moderately stringent criterion requiring at least three consecutive telomeric repeats (each repeat being a permutation of CCCTAACA), HiFi reads with telomeric sequences at both ends predominantly clustered around a length of ∼20 kb (Fig. 1c). After genome annotation, we obtained 27,571 protein-coding genes (PCGs) for *B. undulans*. Completeness assessment of the assembly revealed that 95.4% of the universal orthologs in the BUSCO alveolata_odb10 database were present in *B. undulans*, supporting the high quality of the genome assembly and the completeness of the annotated gene set (Fig. S2). Among the predicted PCGs, 20,654 PCGs (74.9%) could be annotated through at least one of the following protein-related databases: the NCBI non-redundant protein database (NR), the Swiss-Prot protein database, the protein families database (Pfam) and the Gene Ontology (GO) database (Fig. 1d). Among the total PCG set, 23,423 genes (85.0%) were transcriptionally supported, of which 17,839 could be functionally annotated. Notably, among the 4,148 non-transcribed genes, 2,815 could be functionally annotated (Fig. 1e).

**Figure 1:**
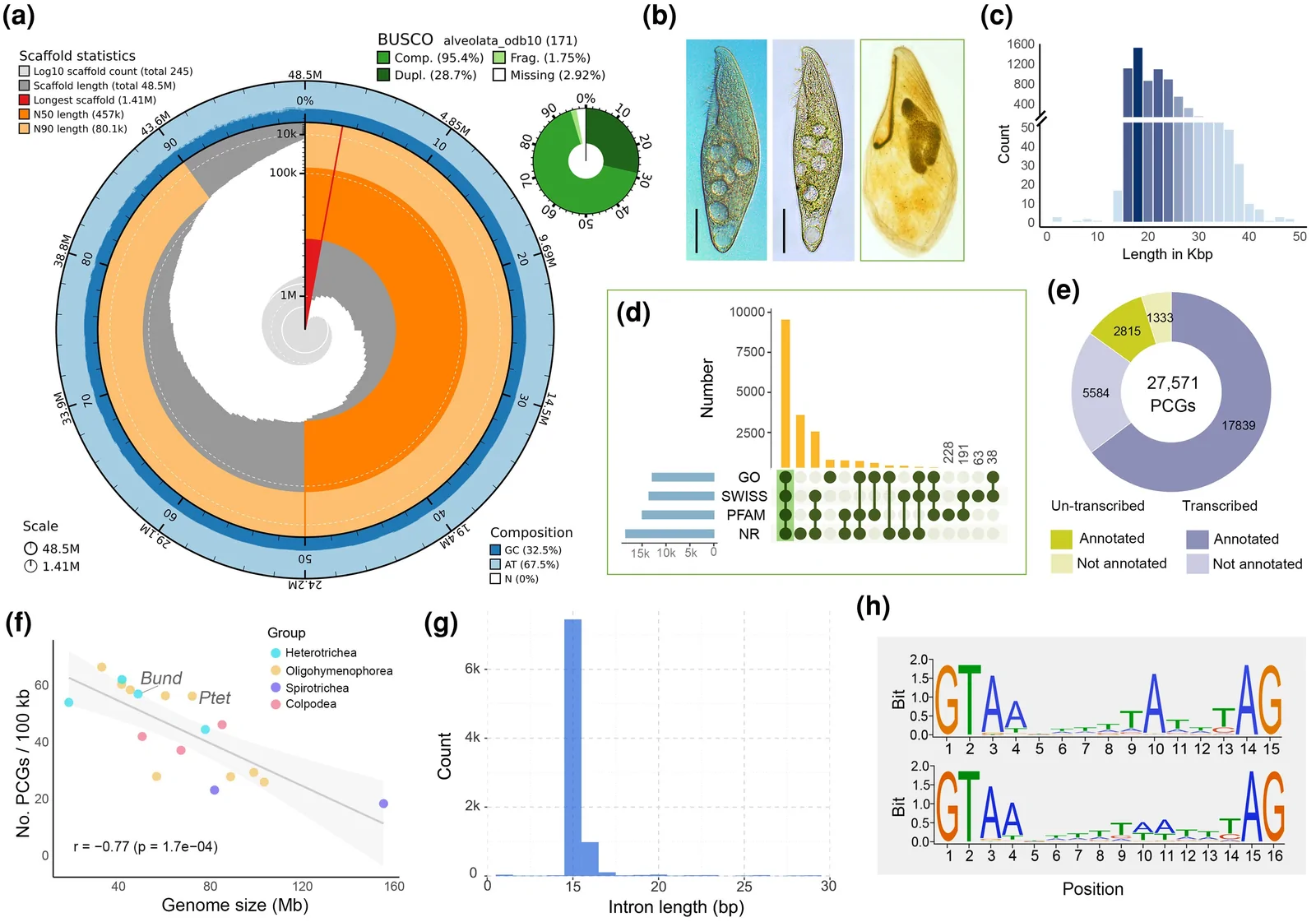
Global statistics for the assembly and annotation of the *B. undulans* genome. a) The BlobToolKit Snailplot summarizes N50 metrics and BUSCO completeness. The circular plot is divided into 1,000 bins, ordered by scaffold size, with each bin representing 0.1% of the 48.5 Mb genome assembly. Scaffold length distribution is shown in dark gray, with the radius scaled to the longest scaffold (1.41 Mb). Orange and pale-orange arcs mark the N50 and N90 scaffold lengths (457 and 80.1 kb, respectively). A pale gray logarithmic spiral indicates the cumulative scaffold count, with white lines marking successive orders of magnitude. The outer blue and pale-blue bands display the GC and AT content across the same bins. In the top right, a BUSCO summary (based on the alveolata_odb10 protein set) shows the proportion of complete, fragmented, duplicated, and missing genes. b) Photomicrographs of *B. undulans* from life and after protargol staining. Scale bars: 50 μm. c) The length distribution of telomeric reads in HiFi library of *B. undulans*. d) The UpSet plot shows the number of genes annotated by different databases. e) Donut chart summarizing the number of transcribed and functionally annotated genes in *B. undulans*. f) Scatter plot illustrating the relationship between genome size and gene density. The *P*-value was derived from the Pearson correlation coefficient analysis. Ptet, *Paramecium tetraurelia*; Bund, *Blepharisma undulans*. g) Intron length distribution in *B. undulans*. h) Highly conserved 15 and 16 bp intron motifs in *B. undulans*. The *y*-axis represents bits, which is based on Shannon information theory and measures the degree of uncertainty reduction at a position.

With a gene density of 56.85 PCGs/100 kb, *B. undulans* exhibits one of the highest coding densities among published ciliate macronuclear genomes (Fig. 1f). This compact genome organization, characterized by both reduced size and elevated gene density, suggests that *B. undulans* has undergone significant genomic streamlining in its macronuclear genome.

In this genome, we identified 9,402 extremely short spliceosomal introns, of which 7,449 (79.2%) were 15 bp and 983 (10.5%) were 16 bp in length (Figs. 1g and S3). Motif enrichment analysis of these two predominant intron types revealed that 15 bp introns harbor a conserved “GTAAWWTTTATWTAG” motif, characterized by a highly conserved adenosine at the 10th position (Fig. 1h). Comparisons with other heterotrich ciliates indicate that these short introns are well conserved and likely dominated in the common ancestor of Heterotrichea (Fig. S4).

### Investigation of intron diversity in ciliates

We demonstrated the phylogenetic relationships among 22 species by constructing a maximum-likelihood (ML) phylogenetic tree based on 1,145 single-copy orthologous proteins. The Intramacronucleata, which represents the majority of ciliates in this study, and the deep-branching subphylum Postciliodesmatophora (represented by the class Heterotrichea) each form monophyletic groups and are sister clades to each other. Using previously developed methodology (Csűrös 2008, 2011), we reconstructed the evolutionary history of introns from 22 representative genomes. To assess the reliability of ancestral intron reconstruction, we calculated the coefficient of variation (CV; standard deviation/mean) of the inferred intron numbers at each ancestral node based on 1,000 bootstrap replicates. Higher CV values indicate lower reconstruction reliability. Branch color intensity in Fig. 2a represents the CV at each ancestral node. Our reconstruction revealed that the last common ancestor of ciliates possessed an intron density of 3.86 introns/CDS kb—higher than that observed in any extant ciliate lineage (maximum 2.87 introns/CDS kb in *Pseudocohnilembus persalinus*), which stands in sharp contrast to the extremely low density in heterotrich ciliates (<0.10 introns/CDS kb) (Figs. 2a and S5; Table S2).

**Figure 2:**
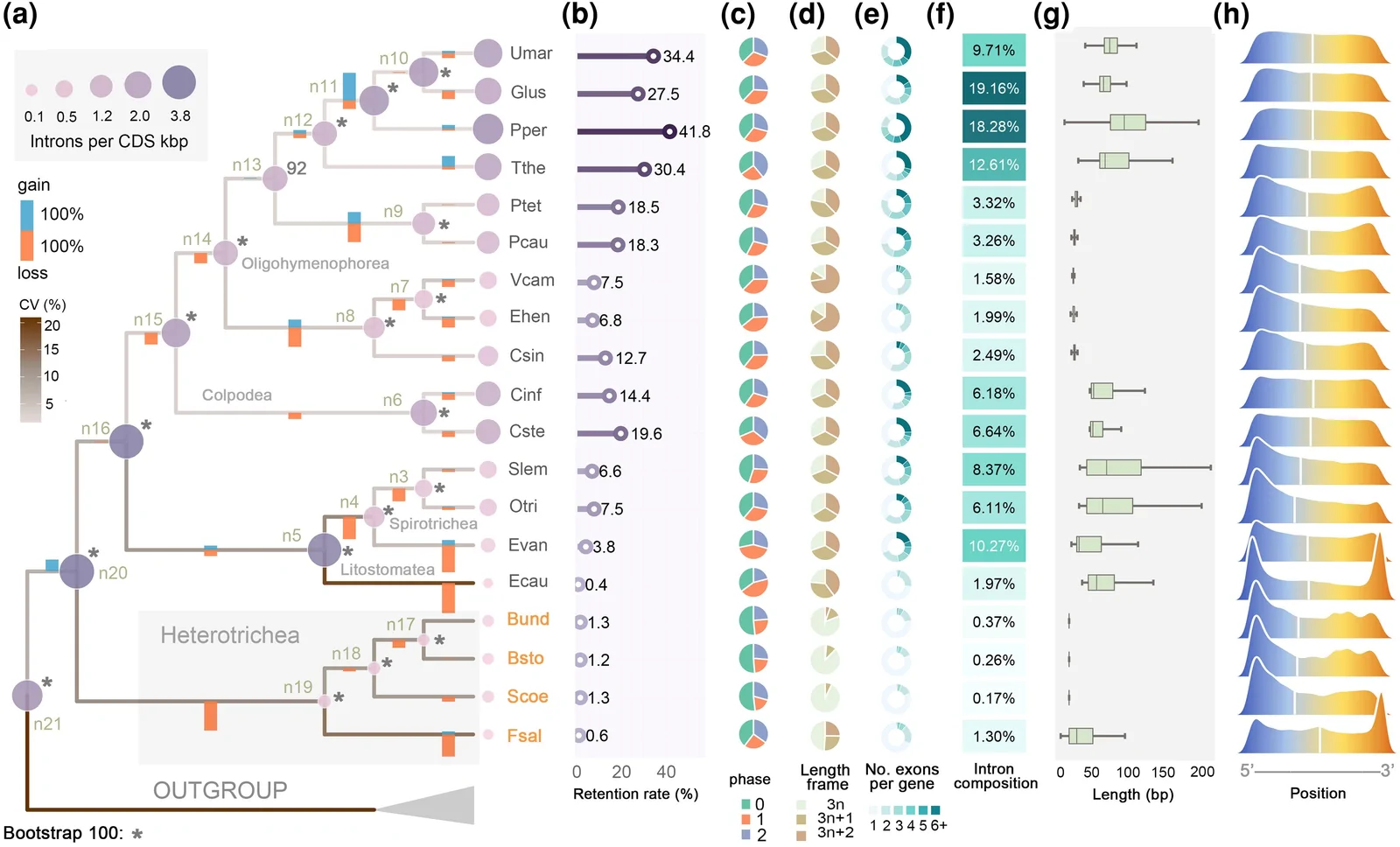
Reconstruction of ancestral introns and structural landscape of introns in ciliates. a) Ancestral introns were inferred from 1,145 orthologous gene sets across 22 species (including three outgroup taxa) using a Markov model that incorporates site-specific rate variation and branch-specific rates of intron gain and loss. The size and color of circles at each node represent the inferred intron density. The rectangles along the branches represent the proportion of intron gains (blue) and losses (orange) relative to the most recent common ancestor. Branch colors represent the CV of intron reconstruction results at each node, calculated based on 1,000 bootstrap replicates. *n* + number denotes node IDs. Bsto, *Blepharisma stoltei*; Bund, *Blepharisma undulans*; Cinf, *Colpoda inflata*; Csin, *Campanella sinica*; Cste, *Colpoda steinii*; Ecau, *Entodinium caudatum*; Ehen, *Epistylis hentscheli*; Evan, *Euplotes vannus*; Fsal, *Fabrea salina*; Glus, *Glauconema* sp.; Otri, *Oxytricha trifallax*; Pcau, *Paramecium caudatum*; Pper, *Pseudocohnilembus persalinus*; Ptet, *Paramecium tetraurelia*; Scoe, *Stentor coeruleus*; Slem, *Stylonychia lemnae*; Tthe, *Tetrahymena thermophila*; Umar, *Uronema marinum*; Vcam, *Vorticella campanula*. b) The proportion of ancestral introns retained in each extant ciliate. c) Pie charts showing the distribution of intron phases (phase 0, phase 1, and phase 2) in each ciliate. d) Pie charts showing the proportion of introns with lengths divisible by 3 (3*n*), 3*n* + 1, and 3*n* + 2 in each ciliate. e) Distribution of genes by the number of exons they contain, shown as a proportion of total genes. f) Proportion of intronic bases in the whole genome. g) Intron length distribution. h) Ridge plot showing the distribution of introns’ relative positions along genes for each ciliate species, from left to right representing the 5′ end to the 3′ end.

At a broad level, intron evolution across ciliate lineages was dominated by intron loss, although the degree of loss varies substantially among different taxonomic groups and evolutionary nodes. For example, the inferred evolutionary trajectory of introns indicated that no substantial intron loss or gain occurred from the ciliate ancestor to the Intramacronucleata lineage, while the common ancestor of the subphylum Postciliodesmatophora underwent massive intron loss after its divergence, with almost no observed intron gain. This shaped the intron-poor genomic architecture characteristic of heterotrich ciliates (Figs. S5 and S6). Notably, ciliate diversification was accompanied by pervasive intron loss, with only limited instances of substantial intron gain (e.g. at node 11 and following the divergence of *Tetrahymena thermophila*). Heterotrich ciliates have retained only ∼1% of the introns present in the common ancestor of ciliates. In contrast, *P. persalinus* has conserved over 40% of these ancestral introns. Additionally, we observed that both peritrich (node 8) and spirotrich ciliates (node 4) exhibit relatively low levels of ancestral intron retention. The endosymbiotic rumen ciliate *Entodinium caudatum* has also experienced extensive intron loss, retaining merely 0.4% of the ancestral introns (Fig. 2b).

Furthermore, we analyzed the phase distribution of introns across all ciliates we used. Introns are categorized into three phases according to their positions relative to codons: phase 0 (between codons), phase 1 (after the first nucleotide of a codon), and phase 2 (after the second nucleotide of a codon). In general, at the interspecific level, closely related species maintained similar phase distributions. Among most ciliates in Intramacronucleata, intron phases are distributed quite evenly. However, a few species have an excess of phase 1 introns, such as *E. caudatum* and *Euplotes vannus*. In contrast, heterotrich ciliates show a strong bias toward phase 0 introns. This is especially evident in *Blepharisma* spp. and *S. coeruleus*, where over 50% of introns belong to phase 0 (Fig. 2c). At the intraspecific level, we found that in some ciliates, intron phase is associated with intron position within genes, and different species exhibit distinct phase preferences. For example, in *T. thermophila*, introns occurring later in the sequence (i.e. those positioned closer to the 3′ end of a gene) are more likely to be inserted in phase 2 and tend to avoid phase 1, whereas in peritrich ciliates, the opposite pattern is observed. In contrast, in certain ciliates such as those in the class Spirotrichea, intron phase appears to be independent of intron position within genes (Fig. S7).

In heterotrich ciliates, the majority of genes (68.3% to 82.0%) lack introns entirely. Intron-containing genes are predominantly single-intron genes (Fig. 2e), with length distributions consistently peaking at 15 bp (Fig. 2g). With the notable exception of heterotrich ciliates, most other lineages exhibited a particularly low occurrence of 3*n* introns (introns with lengths divisible by three) (Fig. 2d). *Blepharisma* spp. and *S. coeruleus* displayed highly constrained intron length distributions, and *Paramecium* spp. together with three peritrich species showed similarly narrow patterns (Fig. 2g). Introns constitute only 0.17% to 1.3% of the genomic sequence in heterotrich ciliates, contrasting sharply with oligohymenophoreans such as *P. persalinus* and *Glauconema* sp., in which introns account for nearly 20% (Fig. 2f).

Differences were also observed in the positional distribution of introns within genes across genomes. *Blepharisma* spp. and *S. coeruleus* exhibited strong 5′-biased intron localization, though *Blepharisma* spp. displayed an unusual multimodal distribution near the 3′ end. *Fabrea salina* showed a markedly distinct pattern, with extreme 3′ enrichment. Broadly, ciliates with higher intron densities exhibited more uniform intron distributions, whereas those with lower densities showed pronounced 5′ bias (with the exception of *Fabrea*) (Fig. 2h).

### Dynamics of intron gain and loss in the evolution of exon-intron architecture

We further investigated how intron loss and gain shaped the diversity landscape of ciliate introns. First, based on a selected set of orthologous genes, we reconstructed the ancestral intron features of the common ancestor of ciliates. The results revealed that introns in the common ancestor of ciliates predominantly exhibited a phase 0 distribution (∼48%), consistent with the pattern observed in most studied canonical eukaryotes (Csűrös et al. 2011). However, we observed excessive loss of phase 0 introns in multiple subsequent descendant nodes (this proportion reaches as high as 59% in *T. thermophila*, and exceeds 50% in several nodes, e.g. node 6 and node 11), which may account for the disappearance of the phase 0 intron bias in many extant ciliate species (Fig. S8).

Our reconstruction of intron loss/gain history further demonstrated that most nodes exhibited preferential intron loss at the 3′-end (as observed in the common ancestor of Heterotrichea), supporting the reverse transcription-mediated intron loss hypothesis. This 3′-biased pattern can be mechanistically explained by the inherent property of reverse transcription, which often initiates at the poly(A) tail of processed mRNAs and frequently terminates prematurely. Consequently, the resulting cDNA copies are disproportionately enriched for the 3′-terminal regions of the transcript, leading to a higher probability of recombining and replacing the 3′-end of the genomic locus, thereby removing introns located in this distal region (Niu et al. 2005). However, exceptions were noted. For instance, in *E. caudatum*, intron loss events were not enriched at either the 5′ or 3′ ends of genes, but occurred predominantly in the central region (Fig. S9). This loss pattern likely directly explains the observed enrichment of introns at both gene termini in this species (Fig. 2h).

Interestingly, *F. salina* exhibited a distinct intron positional distribution compared with other heterotrich ciliates, losing the characteristic 5′-end enrichment while showing pronounced 3′-end accumulation. This pattern aligns with its preferential loss of 5′-proximal introns after divergence from other ciliates (Figs. S9 and S10).

*Blepharisma* species, on the other hand, displayed a unique multimodal intron distribution pattern not observed in other ciliates. While this phenomenon could not be fully explained by intron loss/gain in conserved genes, we found that species-specific newly acquired genes in *Blepharisma* exhibited pronounced enrichment of introns in the central regions of genes, resulting in multiple peaks along the gene body (Fig. 6m). This suggested that introns in novel genes may contribute to shaping the overall intron architecture through distinct distribution patterns.

### Selective retention of tiny introns shapes a molecular safeguard in *Blepharisma*

We proposed that the unique intron features observed in heterotrich ciliates have been shaped by selective pressures. Previous studies (Jaillon et al. 2008; Bondarenko and Gelfand 2016) and our new data consistently indicate that 3*n* introns (introns with lengths divisible by three) are generally disfavored across most ciliates (Fig. 3a). In contrast, heterotrich ciliates predominantly retain introns of precisely 15 bp in length (Fig. 2g). We initially questioned whether the extreme preference for 15 bp introns and the overrepresented phase 0 distribution in heterotrich ciliates might be correlated. Chi-square tests demonstrated a significant association between 3*n* intron status and intron phase distribution in the two heterotrich ciliates, *B. undulans* (χ^2^ = 91.65, df = 4, *P* = 5.87e−19) and *S. coeruleus* (χ^2^ = 69.26, df = 2, *P* = 9.14e−16). By contrast, no such association was observed in *T. thermophila* (χ^2^ = 2.27, df = 4, *P* = 0.686), *Vorticella campanula* (χ^2^ = 2.56, df = 4, *P* = 0.634), or *Stylonychia lemnae* (χ^2^ = 3.90, df = 4, *P* = 0.419). Although *Paramecium tetraurelia* yielded a marginally significant result (χ^2^ = 9.76, df = 4, *P* = 0.045), the extremely small effect size (Cramér's *V* = 0.007) suggests that this marginal significance is likely attributable to the large sample size rather than any biologically relevant association. Validation analyses revealed that in other ciliates, there is no detectable association between whether intron lengths are multiples of three and their phase distribution. In heterotrich ciliates, 3*n* introns are preferentially distributed in phase 0 and phase 2 (Fig. 3b, Table S3).

**Figure 3:**
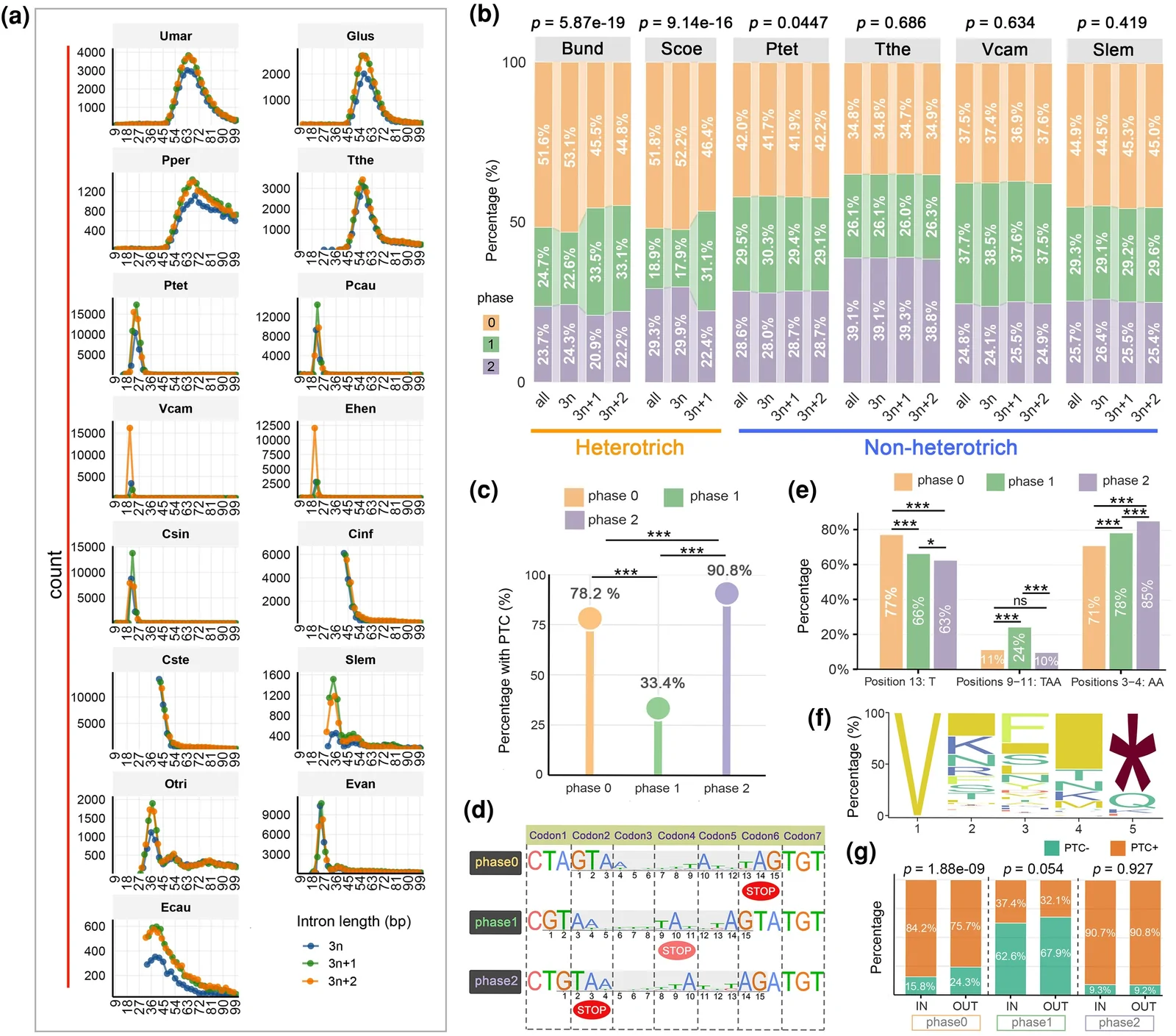
Selective constraints and functional implications of intron phase bias in *B. undulans.* a) Intron length distribution of non-heterotrich ciliates, grouped by divisibility by three, with the *x*-axis limited to lengths below 100 bp. b) Distribution of intron phases across different intron length categories (3*n*, 3*n* + 1, 3*n* + 2) in representative ciliates. The “all” groups include introns of all lengths. c) The proportion of 15 bp introns carrying PTCs across different phases in *B. undulans*. d) Simulation of 15 bp intron retention across three phases. The gray background indicates the motif enrichment results of 15 bp introns. The two inserted consecutive codons were CTA and TGT. The “STOP” labels indicate the codon primarily responsible for generating a PTC. e) Grouped bar plot showing distinct nucleotide compositions contributing to PTC formation among 15 bp introns of different phases. Statistical significance was assessed using the Fisher's exact test. *P < 0.05. **P < 0.01. ***P < 0.001. f) Motif enrichment of translated sequences from 15 bp phase 0 introns under the assumption of intron retention. g) Proportion of PTC-containing introns inside and outside protein domains.

Taking the most abundant 15 bp introns in *B. undulans* as an example, we grouped them by phase and observed distinct frequencies of premature termination codon (PTC) incorporation: phase 0 and phase 2 introns showed significantly higher PTC frequencies (78.2% and 90.8%, respectively), while phase 1 introns had lower PTC occurrence (33.4%; Fisher's exact tests, *P* < 0.001) (Fig. 3c). We further simulated intron retention events for introns of each phase. For the disfavored phase 1 introns, retention would most frequently (66.6%) lead to the incorporation of six random amino acids, with only a limited chance that a TAA stop codon could form at positions 9 to 11. In contrast, retained phase 2 introns have a high probability (85%) of forming a stop codon (TAA) at positions 2 to 4, whereas retained phase 0 introns frequently (77%) generate a stop codon at positions 13 to 15. Specifically, when the third-to-last base is thymine (T), together with the conserved AG acceptor site produces a TAG stop codon (Fig. 3d).

Furthermore, T was notably more frequent at the 13th position in phase 0 introns (77%) than in phase 1 (66%) and phase 2 introns (63%) (Fisher's exact tests, *P* < 0.001). Similarly, the proportion of consecutive adenines (A) at positions 3 to 4 in phase 2 introns was higher than in other phases (85% vs. 78% or 71%) (Fisher's exact tests, *P* < 0.001). Although still relatively low overall, phase 0 introns also showed an increased tendency to form a TAA codon at positions 9 to 11 (24%) (Fisher's exact tests, *P* < 0.001) (Figs. 3e and S11). Retention of PTC-containing introns can lead to premature translation termination, triggering nonsense-mediated decay (NMD) to eliminate mis-spliced mRNAs. Conversely, 3*n* introns lacking PTCs—due to their in-frame location and length divisible by three—do not cause frameshift mutations (Fig. 3f). Their short length (mainly 15 bp) also minimizes structural disruption to the encoded protein. In addition, to investigate the existence of the NMD pathway in heterotrich ciliates, we identified conserved orthologs of its core components: UPF1, UPF2, and UPF3 (Table S4). More importantly, our analysis revealed the presence of SMG6L, a protozoa-specific nuclease reported to be essential for NMD in *Tetrahymena* (Tian et al. 2017). The conserved domain architecture of all these proteins suggests that a functional NMD pathway may be present in this lineage (Fig. S12).

Moreover, based on Pfam domain annotations, introns located within protein structural domains exhibit higher PTC frequencies. For example, 15 bp phase 0 introns located within domains exhibited a higher PTC occurrence than those outside domains (84.2% vs. 75.7%, Fisher's exact test, *P* < 0.001), and a similar trend was observed for phase 1 introns (37.4% vs. 32.1%), although this difference did not reach statistical significance (Fisher's exact test, *P* = 0.054) (Fig. 3g), which may reflect stronger evolutionary constraints in these regions. Additionally, PTC-containing introns display significantly lower retention rates (*t*-test, *P* < 0.001) (Fig. S13). Also, 15 bp introns show a lower retention rate than 16 bp introns (*t*-test, *P* = 5.83e−5) (Fig. S14). These may reflect either higher splicing efficiency or rapid degradation of transcripts retaining these introns via the NMD pathway, leading to under-detection in transcriptomic data.

### Comparative analysis of spliceosomal components reveals patterns of conservation and variation in ciliates

Previous studies have observed independent reductions in spliceosomal components in intron-poor genomes across multiple lineages of the eukaryotic tree of life (Fabrizio et al. 2009; Stark et al. 2015; Black et al. 2023; Whelan and Fast 2023). This prompted us to investigate whether the massive intron loss in heterotrich ciliates is accompanied by the loss of spliceosome-related protein homologs. Given the considerable phylogenetic distance between ciliates and well-studied model organisms, building on a previous study (Sales-Lee et al. 2021), we established a comprehensive identification pipeline that integrates BLAST search results, domain architecture information, and protein length variation, while also accounting for potential false negatives arising from incomplete structural annotations. Homologs of human spliceosomal proteins in ciliates were identified and assessed for completeness at multiple confidence levels. The confidence scores derived from this pipeline were highly consistent with experimentally validated presences or absences of various spliceosomal proteins, confirming the reliability of our bioinformatics approach (Table S5).

A total of 194 non-redundant human spliceosome-related proteins, classified into 21 functional categories from the SpliceosomeDB database, were used as the query set. Among these, the five snRNPs that directly constitute the spliceosomal complex, the core Sm proteins essential for spliceosome assembly and stability, and the second-step factors directly involved in the catalytic reaction together represent the core components required for stable spliceosome function (Matera and Wang 2014). The circular heatmap revealed that these core components are highly conserved across all examined ciliates, with the exception of the nonessential protein CD2BP2, which appears to have been lost in the common ancestor of ciliates. In contrast, the two major classes of splicing regulators responsible for alternative splicing—hnRNP and SR proteins—generally exhibited higher proportions of low-confidence matches (i.e. BLASTp hits lacking conserved domains) in ciliates, suggesting reduced conservation, most likely due to domain loss or structural divergence (Fig. 4a).

**Figure 4:**
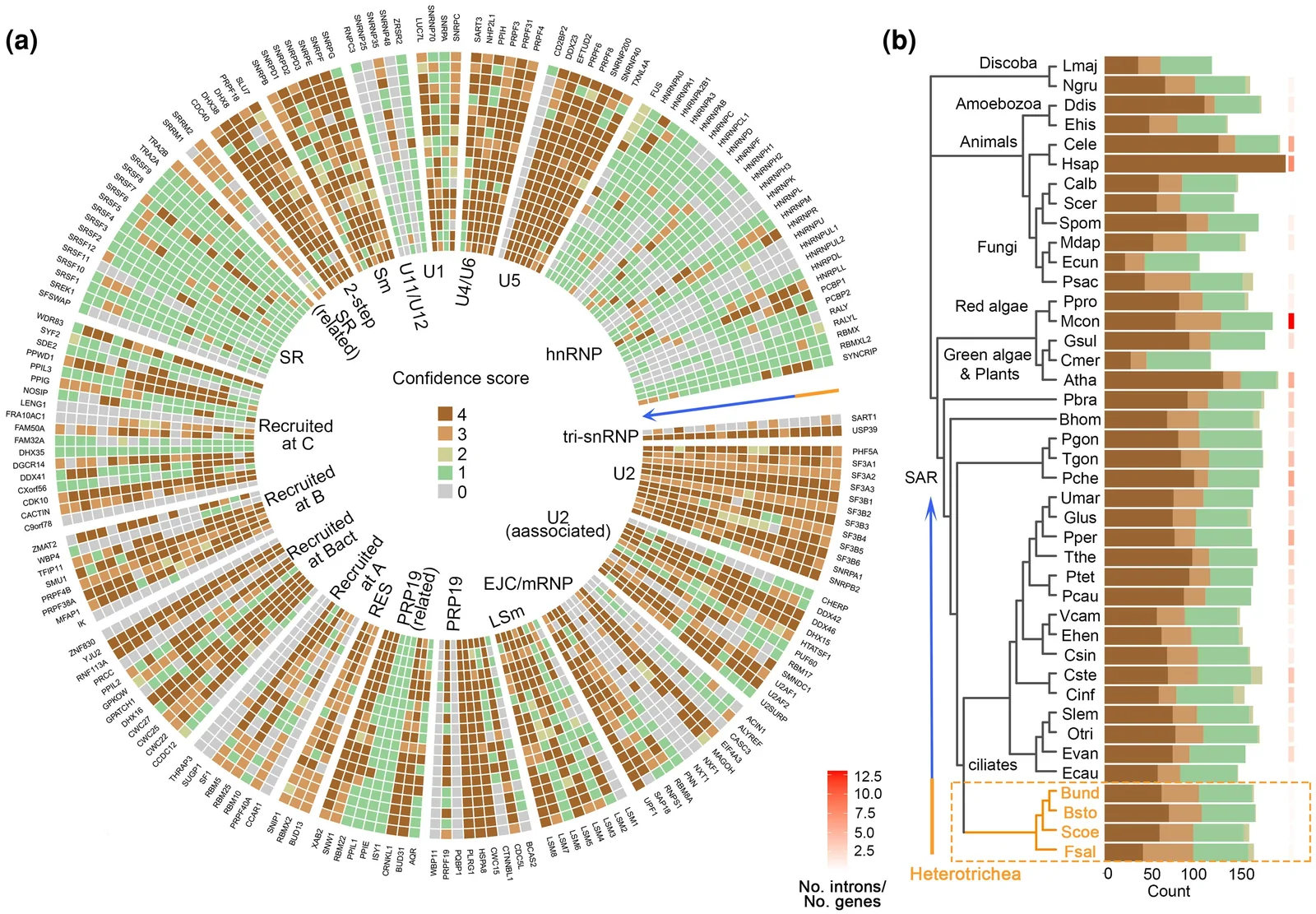
Structural characterization of splicing-related protein components. a) A circular heatmap illustrating the confidence levels of the presence of human splicing-related proteins in ciliates. Each ring represents a species, and proteins are categorized into 21 classes based on the spliceosome database classification. The order of species from the outer to the inner circle corresponds to the sequence indicated by the arrow in (b). Royal blue represents non-heterotrich ciliates, and orange represents heterotrich ciliates. b) Spliceosome-related proteins were retrieved from multiple eukaryotic lineages, including representative intron-poor species. Species selection and eukaryotic phylogenetic relationships were based on recent studies. The phylogenetic relationships of ciliates were inferred in this study, with heterotrichs highlighted in orange. The color scale in the heatmap on the right represents the average number of introns per PCG for each species. *Leishmania major* (Lmaj), *Naegleria gruberi* (Ngru), *Dictyostelium discoideum* (Ddis), *Entamoeba histolytica* (Ehis), *Caenorhabditis elegans* (Cele), *Homo sapiens* (Hsap), *Candida albicans* (Calb), *Saccharomyces cerevisiae* (Scer), *Schizosaccharomyces pombe* (Spom), *Mitosporidium daphniae* (Mdap), *Encephalitozoon cuniculi* (Ecun), *Paramicrosporidium saccamoebae* (Psac), *Pycnococcus provasolii* (Ppro), *Micractinium conductrix* (Mcon), *Galdieria sulphuraria* (Gsul), *Cyanidioschyzon merolae* (Cmer), *Arabidopsis thaliana* (Atha), *Plasmodiophora brassicae* (Pbra), *Blastocystis hominis* (Bhom), *Perkinsus chesapeaki* (Pche), *Toxoplasma gondii* (Tgon), *Plasmodium gonderi* (Pgon).

We selected intron-poor species and their close relatives from multiple eukaryotic lineages for this study (Table S6). Species with extremely intron-poor genomes generally exhibited pronounced spliceosome reduction, consistent with previous reports (Black et al. 2023). In contrast, multiple SAR supergroup species, including ciliates, retained a substantial repertoire of spliceosome-related proteins. Notably, heterotrich ciliates, which have experienced extensive intron loss, did not show appreciable spliceosome reduction. In heterotrichs, we detected 95 to 104 high-confidence (defined as confidence score ≥3, i.e. reciprocal best BLAST hits [Reciprocal Best Hits, RBHs] with human spliceosomal proteins and conserved at the domain level) homologs of human spliceosome-related proteins, a number comparable to that observed in other ciliate lineages (77 to 113) (Fig. 4a, Table S7). The slightly lower numbers in *E. caudatum*, *E. vannus*, and *Colpoda inflata* are likely attributable to the relatively low quality of their genome assembly and annotation (BUSCO completeness of 76% to 89%) (Fig. S2), rather than genuine biological loss. Furthermore, the sequence identities of the putative spliceosomal proteins in heterotrich ciliates to their human orthologs were comparable to those observed in other ciliate lineages (Fig. S15a). It should be noted that, compared with the extremely intron-poor species such as *Saccharomyces cerevisiae* (0.05 introns/gene), the red alga *Cyanidioschyzon merolae* (0.01 introns/gene), and the microsporidian *Encephalitozoon cuniculi* (0.02 introns/gene), the intron abundance in heterotrich ciliates (0.26 to 0.50 introns/gene), despite massive intron loss, remains an order of magnitude higher (Fig. 4b).

To further explore potential structural differences in the spliceosome, we compared the lengths of spliceosomal proteins between heterotrich and other ciliates by functional category. No significant length differences were observed for U2, U5, and Sm proteins (Wilcoxon rank-sum test, *P* > 0.05). However, proteins recruited during the formation of the A complex, B complex, Bact complex, and C complex, as well as SR proteins, exhibited significant length divergence (Wilcoxon rank-sum test, *P* < 0.001). Among SR-related proteins, SRRM1 and SRRM2 in all examined ciliates averaged only 36% and 11% of the length of their human counterparts, respectively. Moreover, no significant difference in length was observed for either protein between heterotrich and non-heterotrich ciliates (Wilcoxon rank-sum test, *P* > 0.05) (Fig. S15b).

U1 snRNA has been shown to be dispensable in intron-poor species (Stark et al. 2015). To assess the presence of spliceosomal snRNAs in ciliates, we systematically scanned ciliate genomes using the Infernal software package (cmbuild and cmsearch). All five major spliceosomal snRNAs (U1, U2, U4, U5, and U6) were identified in all examined species, while no minor spliceosome-specific snRNAs (U11, U12, U4atac, and U6atac) were detected, consistent with the absence of U12-type introns in ciliates. We further integrated representative species from major eukaryotic lineages and systematically compared the sequence features of ciliate snRNAs with those of other eukaryotic groups. In U2 snRNA, which directly participates in spliceosomal catalysis, the branchpoint recognition motif “GUAGUA” is highly conserved across the vast majority of eukaryotes, and was strictly retained in all examined eukaryotic and ciliate species, with only a few nucleotide substitutions observed in two poriferan species, *Amphimedon queenslandica* and *Geodia barretti* (Fig. 5). Notably, this extreme conservation contrasts sharply with the high variability of branchpoint sequences observed in ciliate introns (Fig. S4).

**Figure 5:**
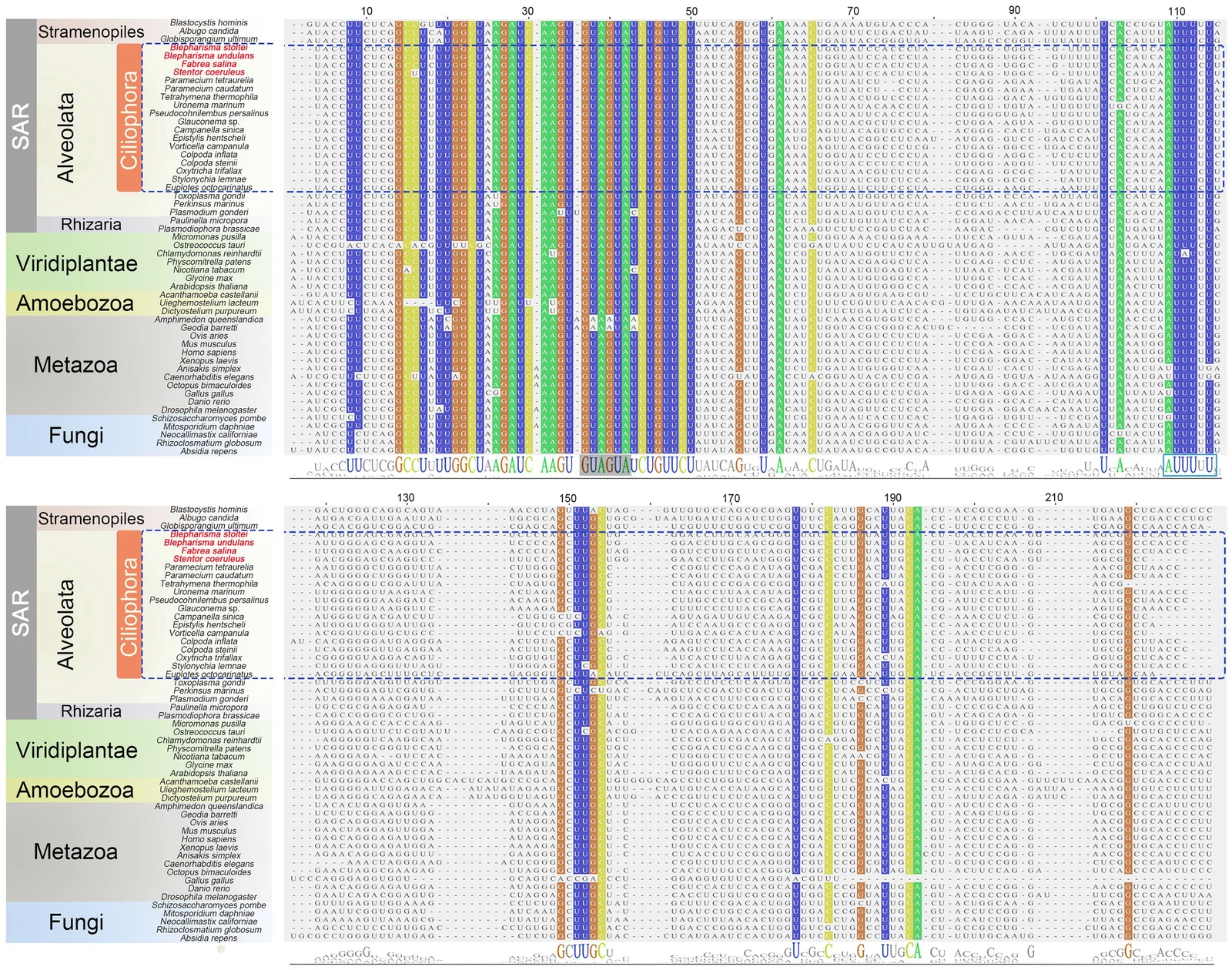
Comparative sequence analyses of spliceosomal snRNAs across major eukaryotic lineages. MSA of U2 snRNAs from diverse eukaryotic lineages. Nucleotides occurring at a frequency >90% at a given position were highlighted in color. Sequences from ciliates were indicated by dashed boxes, and heterotrichs were highlighted in red. Sequence conservation logo was shown below the alignment. The U2 branch point recognition sequence (GUAGUA) was highlighted with a gray background, and the Sm protein–binding motif was outlined in blue.

In addition, all five major snRNAs identified in representative ciliate genomes retained the canonical Sm/Lsm binding motifs. These motifs are critical for the interaction between snRNAs and Sm/Lsm protein complexes, contributing to snRNA structural stability, protection against degradation, and proper spliceosome assembly (Figs. 5 and S16 to S19). Collectively, not only do ciliates retain a complete set of major spliceosomal snRNAs, but their key functional elements are also highly conserved, supporting the stability of the core splicing machinery throughout ciliate evolution.

Principal coordinate analysis (PCoA) based on sequence similarity revealed that, for U1, U2, and U4 snRNAs, ciliates occupy a distinct region of PCoA space separated from other eukaryotic lineages. In contrast, U6 snRNA, which directly participates in the spliceosomal catalytic center, although still forming an independent cluster, was positioned closer to other eukaryotic lineages in PCoA space, reflecting stronger sequence conservation. Notably, metazoans exhibited more pronounced sequence divergence in U5 and U6 snRNAs (Fig. S20).

### Genome evolutionary history and genetic diversity analysis

Although intron evolution is an important mechanism shaping genome structure, interpreting it across different lineages requires a genome-wide evolutionary context. To this end, we included the newly sequenced MAC genome of *B. undulans* in this study to assess its evolutionary history and genetic diversity within ciliates. We compared its genome with publicly available genomes of 21 species (Table S8). In our analysis, *S. coeruleus* was placed as the sister group to *Blepharisma*, and their divergence was estimated at around 404.3 million years ago (Ma). Additionally, we found that these two *Blepharisma* species diverged ∼37.5 Ma. As one of the two representative classes within the subphylum Postciliodesmatophora, Heterotrichea diverged from the Intramacronucleata nearly 1,200 Ma, representing one of the earliest cladogenic events in ciliate evolution. Using CAFE, we further identified 161 expanded and 154 contracted gene families in *B. undulans* (Fig. 6a).

**Figure 6:**
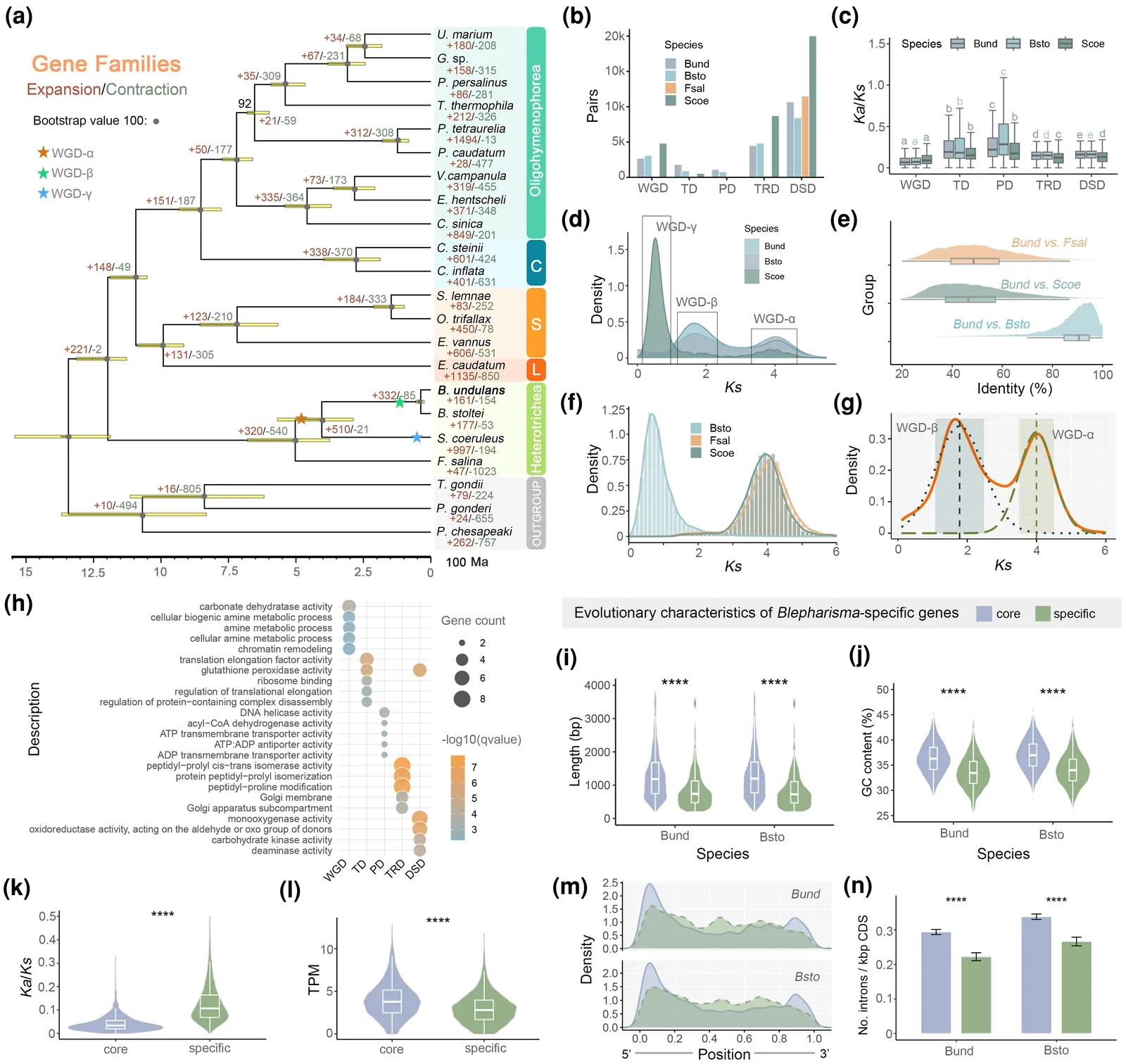
Evolutionary dynamics and genetic innovation in *Blepharisma.* a) A ML phylogenetic tree was reconstructed using 1,145 single-copy orthologous genes from *B. undulans* and 21 species. Node support was assessed with 1,000 ultrafast bootstrap replicates. Divergence times were estimated using Bayesian molecular clock dating, with 95% credibility intervals indicated for each node (in million years ago, Ma). The red and green numbers at each node (including terminal taxa) represent the number of gene families that have expanded and contracted, respectively. C, Colpodea; S, Spirotrichea; L, Litostomatea. b) The number of gene pairs derived from different types of duplication. WGD (whole-genome duplication), TD (tandem duplication), PD (proximal duplication), TRD (transposon duplication), and DSD (dispersed duplication). c) A boxplot illustrating the *Ka*/*Ks* ratio distributions of gene pairs derived from different modes of duplication in heterotrich ciliates. Pairwise comparisons were performed using Dunn's test with BH correction for multiple testing. Different letters above the boxes indicate significant differences between groups (adjusted *P* < 0.05), while groups sharing the same letter are not significantly different (adjusted *P* > 0.05). d) The *Ks* distribution of WGD-derived genes in heterotrich ciliates. e) The comparison of the identity of RBH gene pairs between *B. undulans* and the other three heterotrich ciliates. f) The *Ks* distribution of RBH gene pairs between each pair of species. g) Gaussian mixture models were used to fit the two *Ks* peaks corresponding to WGD events in *B. undulans*, with shaded areas indicating ±1 standard deviation around. h) Functional GO enrichment analysis was performed on genes overlapping between expanded gene families and five modes of gene duplication. Only GO terms with a *P* < 0.01 are shown. For each duplication mode, the top five GO terms with the highest rich factor (the ratio of overlapping genes to the total number of genes annotated to that GO term) are presented to highlight the most significantly enriched biological processes. In the bubble plot, the color of each circle represents the statistical significance of enrichment (measured as −log_10_ of the *q*-value), while the size of the circle corresponds to the number of genes associated with each GO term. *q*-value refers to the false discovery rate-adjusted *P*-value used to control for multiple comparisons. i to n) Evolutionary characteristics of BSGs compared with core genes: gene length (i), GC content (j), *Ka/Ks* ratio (k), expression level (TPM) (l), relative intron position (m) and intron density (n). Light blue-gray indicates core genes, and green indicates BSGs. Statistical significance was assessed using the Wilcoxon rank-sum test. ****P < 0.0001.

We characterized genome-wide gene duplication events in four heterotrich ciliates, classifying them into five categories, i.e. whole-genome duplicates (WGDs), tandem duplicates (TDs), proximal duplicates (PDs), transposon duplicates (TRDs), and dispersed duplicates (DSDs), following previous studies (Qiao et al. 2019; Shi et al. 2020). Our analysis revealed that the two *Blepharisma* species harbored a similar number of WGDs. In contrast, *S. coeruleus* exhibited a substantially higher abundance of WGDs, whereas *F. salina* retained only a negligible number. Notably, both *Blepharisma* species possessed a substantially greater number of TDs and PDs than did the other two heterotrich ciliates (Fig. 6b). Comparative analysis of *Ka*/*Ks* ratios across duplication types indicated that TDs and PDs experienced significantly weaker purifying selection (Dunn's test with Benjamini–Hochberg [BH] correction, adjusted *P* < 0.05), suggesting stronger functional divergence and a greater potential for evolutionary innovation; however, this pattern may also be explained by non-adaptive mechanisms such as genetic drift. Distinguishing between these possibilities will require additional analyses (Fig. 6c, Table S9).

Whole-genome duplication events have been proposed as key evolutionary events driving phenotypic complexity, functional novelty, and ecological adaptation (Wang et al. 2024; Zheng et al. 2025). To investigate WGDs in heterotrich ciliates, *Ks* distributions were characterized in their genomes. To minimize potential noise introduced by tandem duplications or other confounding factors, only gene pairs derived from WGD—specifically, homologous gene pairs located within syntenic regions—were used to calculate *Ks* distributions. At *Ks* = 4.0, both *S. coeruleus* and *Blepharisma* show a *Ks* peak, representing an ancient WGD event (WGD-α) that occurred in their common ancestor. This WGD signature in *S. coeruleus* has become relatively weak. At *Ks* = 1.8, the two *Blepharisma* species share another WGD event (WGD-β), which is absent in *S. coeruleus*. At *Ks* = 0.5, *S. coeruleus* exhibits a prominent *Ks* peak, indicating a WGD event (WGD-γ) that occurred after its divergence from *Blepharisma* (Fig. 6d). The dotplot results of the *Blepharisma stoltei* genome are consistent with the *Ks*-based inference. Furthermore, the dotplot results of *F. salina*, which has almost no WGD-derived gene pairs, similarly do not support the occurrence of a WGD event in this lineage (Figs. S21 and S22). Circos plots showing comparative synteny blocks also support this conclusion (Fig. S23). Additionally, the currently available *S. coeruleus* genome assembly has limited contiguity, making it less suitable for these analyses.

To place these WGDs in a phylogenetic context and assess interspecies divergence, we identified orthologs using the RBH approach. Consistent with their phylogenetic proximity, *B. stoltei* and *B. undulans* exhibited high sequence similarity between orthologous genes, and both *S. coeruleus* and *F. salina* showed substantial genomic divergence from *B. undulans* (Fig. 6e). The *Ks* distributions of orthologous gene pairs between *B. undulans* and other species provided estimates for their divergence times (Fig. 6f). By integrating molecular dating results, we mapped the three WGDs onto the timetree (Fig. 6a), estimating that WGD-α occurred ∼490.3 Ma, WGD-β at 112.5 Ma, and the species-specific WGD-γ at 51.8 Ma. Using Gaussian mixture models, we delineated gene pairs corresponding to each WGD event in *B. undulans* based on the standard deviations of their *Ks* peak distributions (Fig. 6g). Notably, genes derived from the more recent WGD-β exhibited relatively higher differentiation potential (*Ka/Ks* ratios) compared with their ancient WGD-α counterparts (Fig. S24). Functional enrichment analysis revealed no significantly overrepresented biological terms in either gene set, suggesting broad functional conservation post-WGD.

Given that gene duplication is a primary driver of family expansion, we quantified the contributions of different duplication types to gene family evolution in *B. undulans*. Enrichment analysis revealed distinct functional biases among gene duplication types (Table S10). For example, gene families expanded by tandem duplication showed significant enrichment in translational elongation and glutathione peroxidase activity, suggesting roles in oxidative stress response and protein synthesis, while gene families expanded by proximal duplication were enriched for GO terms related to “peptidyl-prolyl” and “Golgi,” implicating their involvement in conformational adjustment of proline-containing peptides and protein processing and trafficking (Fig. 6h). These findings highlight that newly generated tandem and proximal duplications have been important sources of gene family expansion in *B. undulans*, contributing to functional diversification.

### Evolutionary characteristics of *Blepharisma*-specific gene families

Through clustering of orthologous genes, we identified 4,150 gene families exclusively shared between the two *Blepharisma* species (*Blepharisma*-specific genes, BSGs), representing potential genus-specific innovations. Remarkably, 3,919 (94.4%) of these gene families maintained single-copy status in both species.

A comparative analysis against 2,833 conserved core gene families shared across ciliates revealed that BSGs exhibit significantly shorter coding sequences, lower GC content, and lower expression levels compared with core genes (wilcox.test, *P* < 2.2e−16) (Figs. 6i, j, and l). Evolutionary pressure analysis demonstrated that BSGs experience higher *Ka*/*Ks* ratios (wilcox.test, *P* < 2.2e−16), indicating elevated evolutionary divergence potential (Fig. 6k). We detected 14 genes under positive selection among BSGs, while no positively selected genes were identified in the core gene set. Furthermore, BSGs showed distinct spatial distributions of introns within coding regions and reduced intron density compared with core genes (Figs. 6m and n).

Functional annotation challenges were evident, with 33.1% of BSGs lacking reliable functional assignments. GO enrichment analysis revealed that annotated BSGs are significantly enriched in biological processes related to nucleocytoplasmic transport, suggesting their potential involvement in regulating the bidirectional exchange of macromolecules between the nucleus and cytoplasm (Table S11). This process includes, but is not limited to, the export of processed mRNAs for translation and the import of regulatory proteins required for nuclear activities. The rapid shuttling of mRNAs and regulatory proteins between the nucleus and cytoplasm allows cells to swiftly adjust gene expression programs in response to external stimuli (Weis 2003), thereby potentially enhancing the ability of *Blepharisma* to adapt to environmental changes. However, the specific biological significance of this mechanism in *Blepharisma* requires further experimental validation.

## Discussion

### Why choose 15 bp*?*

Introns in heterotrich ciliates exhibit a unique structure—over 80% are precisely 15 bp in length, with phase 0 introns accounting for ∼50%. Rather than reflecting a single deterministic cause, this pattern is likely shaped by the combined effects of multiple evolutionary and mechanistic constraints. Our results suggest that the enrichment of 15 bp introns may reflect a balance among error control, functional preservation, and genomic streamlining.

One possible explanation involves the relationship between intron length and intron phase. The canonical GT-AG splice site dinucleotides can contribute to the formation of PTCs when intron retention occurs, thereby linking splicing signals to nonsense-mediated mRNA decay (NMD). However, this effect differs among intron phases. Phase 1 introns of 3*n* length may be at a selective disadvantage. Their ability to generate PTCs upon retention is relatively limited, and retained phase 1 introns are more likely to introduce short amino acid insertions, which are expected to be deleterious (Behm-Ansmant et al. 2007). Under this assumption, this asymmetric phase selection is suggested to be the fundamental driver behind the biased distribution of 3*n* introns.

However, selection alone does not adequately explain the extreme enrichment of 15 bp introns. Previous studies have indicated that many short 3*n* introns in other ciliates (e.g. *P. tetraurelia*) as well as in diverse eukaryotes, including multicellular organisms like *Arabidopsis thaliana* and *Homo sapiens*, contain abundant PTCs. However, these lineages do not appear to rely primarily on 3*n* introns as a general mechanism for mitigating intron retention errors (Jaillon et al. 2008; Bondarenko and Gelfand 2016). Instead, in *Paramecium* and some ciliate lineages, non-3*n* introns are more commonly implicated in error control through reading-frame disruption following retention (Fig. 3a). Therefore, although the high proportion of PTC-containing introns in heterotrich ciliates is consistent with a potential role in transcript quality control, this observation alone is insufficient to establish PTC-mediated selection as the primary driver of 15 bp intron evolution. Rather, it suggests that different ciliate lineages may have adopted distinct molecular mechanisms to cope with aberrant transcripts while achieving broadly similar functional outcomes.

Beyond selection, mechanistic constraints likely also contribute to the emergence of 15 bp introns. Although short introns have certain disadvantages—for example, the BP region requires adequate flanking sequence for U2 snRNP recognition (Coolidge et al. 1997; Newnham and Query 2001), and very short introns may therefore compromise splicing efficiency, they also present clear advantages. In particular, having fewer and shorter introns may promote genome compaction, potentially reducing DNA replication time and accelerating cell division (Parenteau and Abou Elela 2019). Consequently, 15 bp may approach the lower boundary of intron length compatible with stable spliceosomal recognition and assembly in heterotrich ciliates. Additional shortening would be expected to either reduce splicing efficiency or increase the frequency of mis-splicing events. In this scenario, 15 bp does not necessarily correspond to an evolutionarily optimal length, but rather to a minimally feasible length imposed by the splicing machinery. Collectively, we propose that the enrichment of 15 bp introns most likely reflects an equilibrium between selective benefit and physical/structural constraints, rather than being fully attributable to a single mechanism.

Furthermore, we observed that introns lacking PTCs are rarely located within key protein domains. This spatial avoidance likely minimizes the harmful effects of random peptide insertions into core functional regions, suggesting that intron positioning may be constrained by the need to preserve protein functional integrity.

Overall, our results suggest that the enrichment of 15 bp introns in heterotrich ciliates should not be simply interpreted as a single adaptive trait optimized by natural selection. Rather, it more likely represents a stable evolutionary state emerging from the long-term interplay of selective forces and molecular constraints. Furthermore, the retention of these introns—leading to NMD—may itself serve as a mechanism for gene expression regulation. This framework provides a new perspective for understanding the origin and maintenance of ultra-short introns, as well as how genomes achieve a balance between functional demands and structural constraints.

### Deep divergence of the ciliate intron landscape revealed by genomic analyses

Ciliates are a well-defined and rapidly evolving clade of single-celled eukaryotes with exceptional diversity in exon-intron organization (Gao et al. 2017; Jiang et al. 2024). As a lineage phylogenetically independent of metazoan, fungi, and plants, they provide an underexplored comparative system with substantial phylogenetic depth and wide variation in intron content, ranging from intron-rich to intron-poor genomes, enabling robust evolutionary analyses of the forces shaping intron evolution across eukaryotes (Rogozin et al. 2003; Csűrös et al. 2011; Yenerall et al. 2011; Lim et al. 2021).

Early investigations into ciliate introns have primarily emphasized basic features such as intron length and density (Bondarenko and Gelfand 2016; Nuadthaisong et al. 2022). However, the lack of high-quality genome assemblies in previous decades likely resulted in a substantial underestimation of the structural diversity of introns in this phylum. By employing a broader taxonomic sampling and utilizing high-quality genome data from multiple ciliate lineages, our study provided a more comprehensive and refined characterization of the intron landscape in ciliates. As anticipated (Glick et al. 2024), closely related ciliate species tend to exhibit similar intron structural profiles. Nevertheless, our findings revealed that the structural divergence of introns among different ciliate groups is far more pronounced than previously recognized. This diversity was reflected in multiple dimensions, including intron length distributions, intron density, splicing site motifs, phase biases, and positional arrangements within genes (Fig. 2).

In addition, we observed intriguing lineage-specific patterns. In some species, such as *T. thermophila*, a clear relationship exists between intron phase and its sequential order: a marked shift toward phase 2 and away from phase 1 is evident when comparing, for example, the tenth intron to the first. Conversely, in other ciliates, no apparent relationship between intron phase and sequential order was detected (Fig. S7). These contrasting patterns suggest that different ciliate lineages may have undergone independent intron structural refinements.

Moreover, intron positional distribution within genes appears to be associated with species-specific ecological strategies and genomic features. For example, the endosymbiotic rumen ciliate *E. caudatum* breaks the typical bias of intron loss at gene termini, exhibiting a unique pattern in which introns are enriched at both ends of genes. 3′-end introns can potentially impact on transcription termination, mRNA stability and cellular localization (Baurén et al. 1998; Andersen et al. 2013; Choquet et al. 2025). The selective retention of 3′-end introns may facilitate rapid regulation of gene expression in highly fluctuating environments. Another species, *F. salina*, which possesses a highly streamlined genome, shows preferential loss of 5′-end introns, resulting in a directional accumulation of introns toward the 3′ end. These observations suggest that the dynamic positioning of introns may be linked to lineage-specific survival strategies, such as endosymbiosis, and to overall genomic architecture, such as extreme genome reduction.

Through ancestral intron reconstruction, we demonstrated that intron evolution in ciliates has followed lineage-specific trajectories. The most extensive intron loss occurred at the ancestral node of the heterotrich ciliates, with no subsequent large-scale intron gains observed in later evolutionary stages. The ancestral ciliates exhibited a high proportion of phase 0 introns, similar to patterns seen in other eukaryotes (Csűrös et al. 2011). The subsequent excessive loss of phase 0 introns observed in multiple descendant nodes (with phase 0 introns accounting for >50% of lost introns in some nodes) suggests that biased intron loss events may have driven the attenuation of phase 0 intron predominance in many extant ciliate species, thereby reshaping their intron phase distribution patterns. Furthermore, the unique multimodal intron distribution observed in *Blepharisma* spp., combined with contrasting intron patterns of genus-specific genes and conserved genes, reveals that intron landscapes are shaped through a multi-layered process. This process is influenced not only by ancestral states and selective intron loss, but also by the emergence of new genes, which are often overlooked in ancestral reconstructions.

### Conservation and divergence of the spliceosomal machinery in ciliates

The widespread intron loss in heterotrich ciliates raises the key question of whether this genomic simplification extends to the spliceosome itself. In this study, we systematically examined the retention and potential reduction of spliceosome-related components in ciliates, particularly in heterotrichs that have experienced extensive intron loss. Our results demonstrate that despite drastic intron reduction, the core spliceosomal components remain largely intact in heterotrich ciliates, suggesting that intron loss and spliceosomal protein reduction are not necessarily coupled in this lineage (Fig. 4b). This finding contrasts with the general trend observed in multiple eukaryotic lineages, where extremely intron-poor species such as microsporidia, the red alga *C. merolae*, and *S. cerevisiae* have undergone varying degrees of spliceosomal protein and snRNA loss, with some lineages even losing core components such as U1 snRNA, indicating a strong correlation between intron depletion and spliceosome reduction (Stark et al. 2015; Sales-Lee et al. 2021). In our study, heterotrich ciliates maintained a complete core splicing machinery despite extensive intron loss. This observation indicates that intron loss does not invariably lead to co-occurring spliceosome degeneration, and different eukaryotic lineages may follow distinct evolutionary trajectories with respect to spliceosome evolution.

One possible explanation is that, although heterotrich ciliates have experienced substantial intron loss, their intron abundance remains an order of magnitude higher than that of typical intron-poor species known to exhibit significant spliceosome reduction. Thus, compared with these extremely intron-depleted species, heterotrich genomes still retain a considerable number of introns, and their splicing processes likely continue to require a fully functional spliceosome. This result suggests that the key determinant of spliceosome reduction may not be intron loss per se, but rather that the selective pressure for maintaining a complex splicing machinery only diminishes when intron density falls below a certain critical threshold. However, this hypothesis awaits further validation through comparative genomic analyses incorporating a broader range of species with varying intron densities.

Notably, despite the high conservation of core spliceosomal components, distinct evolutionary patterns emerge across different functional categories. Core proteins constituting the five snRNPs, Sm proteins, and the second-step factors are highly conserved across all examined ciliates, whereas SR proteins and hnRNPs, which are primarily involved in splicing regulation, generally exhibit reduced conservation (Fig. 4a). Meanwhile, proteins involved in intermediate spliceosomal assembly steps (such as the A, B, Bact, and C complexes), as well as certain SR-related proteins, show moderate length and structural divergence among different ciliate lineages, suggesting possible adaptations to varying intron lengths or splicing kinetics (Fig. S15b). However, since our identification of spliceosomal components was based on human spliceosome protein databases, we cannot exclude the possibility that ciliates possess lineage-specific essential splicing factors. Further experimental validation, such as mass spectrometry and structural electron microscopy, would facilitate a better understanding of the ciliate splicing machinery. These differences indicate that core components directly involved in catalysis and spliceosome assembly experience stronger purifying selection, whereas peripheral regulatory modules primarily engaged in splicing regulation and protein-protein interactions exhibit greater structural plasticity. Similar modular conservation patterns have been reported in comparative spliceosome studies across other eukaryotes, suggesting that different spliceosomal subcomplexes are subject to varying degrees of evolutionary constraint (Fabrizio et al. 2009; Black et al. 2023; Whelan and Fast 2023).

Our comparative analysis of snRNAs further supports this view. All ciliates retain the complete set of five major spliceosomal snRNAs, maintaining key functional elements including Sm/Lsm binding sites and the branchpoint recognition motif of U2 snRNA (Figs. S16 to S19). The GUAGUA motif in U2 snRNA, which is involved in branchpoint recognition, is highly conserved across diverse eukaryotic lineages, whereas branchpoint sequences in ciliate introns display considerable diversity (Figs. 5 and S4). This phenomenon suggests that although the *cis*-acting elements of ciliate introns have undergone substantial evolution, the core snRNA responsible for their recognition remains highly stable, and the recognition mechanism likely relies on conserved RNA structures and cooperative interactions with spliceosomal proteins, rather than solely on the branchpoint sequence itself.

In summary, our findings reveal a decoupling between core splicing components and regulatory splicing factors in ciliates. Heterotrich ciliates have maintained an efficient and streamlined splicing system by retaining the catalytic core while reducing the structural complexity of regulatory components—an exemplary case of evolutionary simplification without loss of function.

### Gene duplications act as central drivers of genome evolution in heterotrich ciliates

Here, we report a new reference MAC genome for *B. undulans*, a representative species of the heterotrich ciliates. Despite its compact 48.5 Mb genome size, it encodes 27,571 PCGs, demonstrating a notably high gene density. Gene duplication has been proposed as a key evolutionary driver in the genus *Blepharisma*. To investigate its impact, we conducted a systematic genomic analysis of heterotrich ciliates, which revealed the significant contributions of diverse gene duplication events to their evolutionary history.

At the whole-genome level, we identified three distinct WGD events: the ancient WGD-α (∼490 Ma) provided the genetic foundation for the early divergence of the genera *Blepharisma* and *Stentor*; the more recent WGD-β (∼112 Ma) occurred specifically within *Blepharisma*; while WGD-γ (∼52 Ma) was unique to *S. coeruleus*. In both the WGD-α and WGD-β, species diversification occurred shortly after the duplication events, suggesting a close association between WGDs and speciation within Heterotrichea—an observation also reported in the first ciliate genus in which WGDs were identified, *Paramecium*. Notably, genes derived from the more recent WGD-β exhibited higher *Ka/Ks* ratios compared with their ancient WGD-α counterparts, indicating stronger differentiation potential during evolution. This pattern is consistent with observations in *P. tetraurelia* as well as in other eukaryotic lineages (Aury et al. 2006; Brunet et al. 2006; Wang et al. 2024; Ni et al. 2025).

Beyond whole-genome duplications, *Blepharisma* spp. exhibited a higher proportion of tandem and proximal duplications. These duplication types experienced relatively weaker purifying selection, suggesting their potential importance as sources of novel functions. Gene families expanded through tandem duplication showed significant enrichment in translational elongation and glutathione peroxidase activity, with the latter being potentially involved in the oxidative stress response, while proximally duplicated genes were primarily associated with Golgi-related functions and protein folding processes. Tandem and proximal duplicates have been observed across genomic analyses of multiple plant and metazoan lineages to exhibit significantly higher *Ka/Ks* ratios, suggesting that they may represent a widespread mechanism of functional innovation in eukaryotes (Qiao et al. 2019; Lu et al. 2021).

Collectively, these findings illustrate the multi-layered contributions of gene duplication to ciliate evolution: whole-genome duplications provide the raw material for macroevolution and may be closely linked to species diversification, while small-scale tandem and proximal duplications drive adaptive expansion of gene families through rapid functional diversification. The complementary effects of different duplication mechanisms collectively shape the genomic plasticity and environmental adaptability of heterotrich ciliates.

## Materials and methods

### Sample preparation

The collection site of *B. undulans* is located in the Lake Weishan wetland in Jining, China (34°44′47″ N; 117°10′02″ E). Species identification of *B. undulans* was conducted based on live observations and protargol staining method (Fig. 1b), accompanied by the extraction and validation of SSU rDNA sequences. Cultivation was carried out using lettuce juice medium at a concentration of 1×, and the organisms were maintained in a constant-temperature incubator at 25 °C. To enhance the nutritional concentration of the medium, lettuce juice pre-inoculated with *Klebsiella pneumoniae* was utilized and cultured at 37 °C for ∼24 h to obtain a more concentrated bacterial solution, serving as the food source for *B. undulans*.

### Whole-genome sequencing and whole-transcriptome sequencing

First, the culture medium of *B. undulans* was filtered using eight layers of sterile gauze to remove lettuce debris, followed by centrifugation at 200 × *g* for 3 min to concentrate the cells. The surface of the organisms was then repeatedly washed with sterile mineral water by centrifuge. Subsequently, the organisms were subjected to starvation treatment for 24 h to digest the DNA ingested from *K. pneumoniae*. DNA extraction was conducted using the conventional phenol-chloroform method followed by ethanol precipitation, while RNA extraction was performed with the RNAiso Plus Kit (Takara, Cat. No. 9109, Japan). Subsequently, the integrity and quality of the extracted nucleic acids were assessed via agarose gel electrophoresis. The concentration of the extracted DNA and RNA was measured using a Qubit fluorometer. Long-read DNA sequencing was performed using the PacBio Sequel II platform, while Illumina DNA and RNA sequencing was performed using the Illumina NovaSeq6000 sequencer for paired-end 150 bp (PE150) sequencing.

### Genome size estimation and genome assembly

Using Illumina sequencing data, the basic features of the genome were assessed, including genome size, heterozygosity, proportion of repetitive sequences, and GC content. Fastp v0.23.2 with default parameters was used to filter out adapter sequences and low-quality reads from the raw short-read data (Chen 2023). Genome size and heterozygosity were evaluated based on k-mer frequency distribution. After filtering, a total of 22.69 Gb of reads were obtained. The frequency distribution of 21-mers was then calculated using Jellyfish v2.2.3 (Marçais and Kingsford 2011), followed by prediction of genome size and heterozygosity using GenomeScope v2.0.1 (Ranallo-Benavidez et al. 2020). PacBio HiFi reads were assembled using Canu v2.1.1 (Koren et al. 2017). Potential genome assembly contamination was detected using FCS-GX v0.5.5 with default parameters and subsequently removed (Astashyn et al. 2024). Then, contigs with GC content higher than 40% were removed. Redundans v2.0.1 (Pryszcz and Gabaldón 2016) and purge_dups v1.2.5 (Guan et al. 2020) were used to remove redundant contigs based on similarity and coverage. To increase the accuracy of the assembly, the contig-level assembly was polished by Pilon v1.2.2 (Walker et al. 2014). Assembly qualities were evaluated by QUAST v5.0.2 (Gurevich et al. 2013). BlobToolKit v4.4.6 (Challis et al. 2020) was used to generate the snail plot, which visualizes the genome assembly quality.

### Gene modeling and annotation

RepeatMasker v4.0.7 (Chen 2004) and RepeatModeler v1.0.8 (Flynn et al. 2020) were used to predict repetitive sequences. The gene models were predicted using a fully automated and parallelized pipeline, egn-ep v1.6.5 (2021-07-05) (http://eugene.toulouse.inra.fr/Downloads/egnep-Linux-x86_64.1.6.5.tar.gz). This pipeline executed probabilistic sequence model training, repeat masking, transcriptome and proteome alignment, and integrated gene model construction within the EuGene v4.3 software (Foissac et al. 2008). The protein sequences used for gene annotation were derived from two closely related species to the subject organism: *B. stoltei* ATCC 30299 (BUSCO: 94.7%) and *S. coeruleus* (BUSCO: 93.0%). The RNA-seq reads were aligned to the reference genome using HISAT2 v2.2.1 (Kim et al. 2019). Transcripts were then assembled using StringTie v1.2.7 (Pertea et al. 2015). tRNAscan-SE v2.0.9 (Lowe and Eddy 1997) was used to identify transfer RNA (tRNA) genes in the *Blepharisma* genome. Functional annotation for all PCGs was performed by mapping their protein sequences to the following databases (InterPro [Blum et al. 2021], SWISS-PROT [Boeckmann et al. 2003], Pfam [Mistry et al. 2021] and GO [Gene Ontology Consortium 2004]) using BLASTp (Boratyn et al. 2013) or HMMER (Potter et al. 2018) with default parameters (-*e*value 1e−5). We also used BUSCO v4.0.4 (Manni et al. 2021) with alveolata_odb10 (2024-01-08) to assess the completeness of the genome annotation.

### Phylogenetic relationship reconstruction

SonicParanoid v2.0.8 (Cosentino et al. 2024) was used to construct orthologous gene families across *Blepharisma* and other ciliate species. The phylogenetic tree was reconstructed based on 1,145 single-copy genes and designated other three alveolates (*Toxoplasma gondii*, *Perkinsus chesapeaki*, and *Plasmodium gonderi*) as the outgroup (Table S12). The selection of orthologous gene datasets was based on three criteria: (i) the gene is present in more than 80% of the selected species, (ii) the gene is present in at least one of the three outgroup species, and (iii) the gene is a single-copy gene in at least 50% of the species. MAFFT v7.505 (Katoh and Standley 2013) was used to align the homologous regions of the single-copy orthologous genes using the L-INS-i strategy. TrimAl v1.4.rev15 (Capella-Gutiérrez et al. 2009) was used to extract conserved sites from multiple sequence alignment (MSA) results. A phylogenetic tree was constructed using IQ-TREE v2.1.4-beta (Minh et al. 2020) with the LG + F + R7 model. The robustness of each branch was assessed through 1,000 bootstrap replicates.

### Analyses of intron features

Custom Python scripts (https://github.com/ZhaoruiZhou/Intron_Evolution_in_Ciliates) were utilized to analyze intron features, including intron position, length, phase, GC content, distribution patterns along genes, their genomic abundance, the fraction of introns divisible by three, and the proportion of genes containing varying numbers of introns.

### Inference of ancestral introns

We inferred ancestral intron content and intron gain and loss dynamics using Malin (2008 April 13 version) (Csűrös 2008), based on 1,145 sets of orthologs from 22 genomes (as described in Phylogenetic relationship reconstruction). The selected protein sequences were aligned, and intron positions were annotated to generate input files for Malin. Additionally, the phylogenetic relationships, derived from multi-gene concatenated tree construction, were provided as input.

An intron presence/absence table comprising 16,571 homologous intron sites was constructed, allowing up to five ambiguous characters per site, which account for missing orthologs and alignment uncertainty and are explicitly handled by the likelihood framework in Malin. Intron evolution was modeled under a ML framework with branch-specific gain and loss rates. We used two discrete rate categories for intron loss and two for intron gain. We applied a rate-variation model, starting from the standard null model, with 1,000 optimization rounds and a likelihood convergence threshold of 0.001.

To assess uncertainty in inferred rate parameters and evolutionary histories, we conducted 1,000 bootstrap replicates in Malin. Variability in inferred intron numbers at ancestral nodes was quantified using the CV (standard deviation/mean). Ancestral intron densities were estimated from the median values across the 1,000 bootstrap replicates. Furthermore, the complete results from the 1,000 bootstrap replicates, together with the original MSAs and other files required to reproduce our analyses, have been deposited in our GitHub repository (https://github.com/ZhaoruiZhou/Intron_Evolution_in_Ciliates).

For comparative analyses, we normalized intron counts to relative density values using *T. thermophila* as a well-annotated reference species. The conversion was calculated as: Relative Intron Density = (Inferred Intron Count × 2.0)/1,000, where 2.0 represents the intron density (introns per kb of CDS) in all *Tetrahymena* genes, and 1,000 corresponds to the total number of inferred introns in *Tetrahymena*'s ortholog set (Table S2).

Only intron site histories (intron loss or gain events) with a posterior probability ≥0.99 were retained for downstream analysis (Table S13). We analyzed the presence and absence of intron sites across each lineage, encompassing both extant taxa and ancestral nodes, and also examined the number of introns shared among extant ciliates and their most recent common ancestor. The R package ggtree v3.10.1 (Xu et al. 2022) was used for visualizing the phylogenetic trees.

### Searches for spliceosomal components

Following established methods from a prior study (Sales-Lee et al. 2021), the search for spliceosomal protein components was based on sequence homology alignment and domain comparison. BLASTp v2.5.0 and tBLASTn v2.5.0 (-*e*value 1e−5) (Boratyn et al. 2013) were used for protein sequence alignment and to identify newly annotated coding regions in the genome. HMMER v3.3.2 (Potter et al. 2018) was employed with default parameters to search for domains in both human spliceosomal proteins and proteins of ciliates. To refine the alignment assessment of human spliceosome-related proteins in ciliates, we adopted a scoring system inspired by prior research (Sales-Lee et al. 2021). For each protein component, we assigned scores based on the following criteria: A score of 4 was given if the protein had a RBH alignment in the corresponding ciliate, domain-level support, and sequence length variation within ±30%. If the first two conditions were met but the sequence length variation exceeded this range, the score was 3. A score of 2 was assigned if there was only sequence-level alignment without domain support. If no BLASTp alignment was found but a tBLASTn alignment was present, the score was 1. Finally, if no alignment was obtained, the score was 0. This scoring system aimed to provide a more nuanced evaluation than traditional alignment algorithms. This analysis was conducted using custom Python scripts available at https://github.com/ZhaoruiZhou/Intron_Evolution_in_Ciliates. A circular heatmap was generated using the R packages (ComplexHeatmap v2.18.0 [Gu 2022] and circlize v0.4.16 [Gu et al. 2014]).

Covariance models for the five spliceosomal snRNAs (U1, U2, U4, U5, and U6) were downloaded from the Rfam database v15.1 (Ontiveros-Palacios et al. 2025) and used as seed models for sequence searches in ciliate genomes using cmsearch from the Infernal package v1.1.4 (Nawrocki and Eddy 2013). For each genome, only the best-scoring hit was retained for downstream analyses. snRNA sequences from other eukaryotic lineages were directly obtained from the Rfam database. MSAs were generated using MAFFT v7.505 with the L-INS-i algorithm (Katoh and Standley 2013), and alignment visualization was performed using Jalview v2.11.5.1 (Waterhouse et al. 2009). PCoA was conducted based on pairwise sequence similarity matrices and implemented using custom R scripts.

### Gene family analysis and divergence time estimation

Divergence time was estimated using the MCMCTree program in PAML v4.6 (Yang 2007) with the main parameters burn-in = 20,000, sample-number = 1,000,000, and sample-frequency = 10. Calibration time nodes were selected based on a previous study (Parfrey et al. 2011). Tracer v1.7.12 (Rambaut et al. 2018) was used to confirm that the MCMC runs had reached convergence, with all effective sample size values exceeding 200. We used CAFE v5.1.0 (Mendes et al. 2021) to infer gene family copy number evolution along the branches of the phylogeny we constructed. ClusterProfiler v4.0 (Wu et al. 2021) was used to identify GO terms enriched in each gene list determined in the above analyses.

### Genome duplication and intergenomic comparison

We used DupGen_finder's DupGen_finder-unique.pl script to identify different patterns of duplicate genes and classify them into five types based on their evolutionary origins, including WGD (whole-genome duplication), TD (tandem duplication, i.e. adjacent duplicate genes), PD (proximal duplication, i.e. duplicate genes separated by no more than 10 genes), TRD (transposon duplication, i.e. duplicate genes composed of ancestral and new gene loci), and DSD (dispersed duplication, i.e. duplicate genes that are neither adjacent nor collinear). Homologous gene pairs were selected using BLASTp v2.5.0 (-*e*value 1e−10) (Boratyn et al. 2013) to choose the top 5 best matches (Qiao et al. 2019). TBtools v2.485 (Chen et al. 2023) was used to generate genomic dotplots and Circos plots. Syntenic blocks were defined as containing more than 5 consecutive collinear genes.

The protein sequences of each duplicate pair were aligned using MAFFT v7.505 (Katoh and Standley 2013) with the L-INS-i mode, and then converted into codon alignments using PAL2NAL (Suyama et al. 2006). Subsequently, a custom Perl script was employed to format the generated codon alignments into AXT format. The nonsynonymous substitution rate (*Ka*) and the synonymous substitution rate (*Ks*) were calculated using the γ-MYN method (an improved version of the Yang-Nielsen method) implemented in KaKs_Calculator v2.0 (Wang et al. 2010). *Ka* and *Ks* were calculated using the codon table 4 for *Blepharisma*, while the standard codon table 1 was used for *Stentor* and *Fabrea*.

### Identification of WGD events and genome synteny

We extracted *Ks* values for WGD gene pairs in *Blepharisma* and *Stentor*, and then constructed their density distributions using ggplot2 v3.5.1 (Wickham 2011). Considering synonymous substitution saturation, *Ks* > 5.0 were excluded from analysis (Vanneste et al. 2013; Li et al. 2016). To identify peaks and standard deviations of *Ks* distributions, we employed Gaussian Mixture Modeling via the mclust v6.1.1 package (Scrucca et al. 2016) to classify gene pairs corresponding to different WGD events.

### Statistical analyses

All statistical analyses were performed in R v4.3.0 (R Core Team 2021). Fisher's exact test, Student's *t*-test (*t*-test), and chi-square test were performed using the built-in R functions. The Wilcoxon rank-sum test was performed using the rstatix v0.7.2 package (Kassambara 2019). Following a significant Kruskal–Wallis test, Dunn's test was performed using the FSA v0.10.1 package (Ogle et al. 2026), with *P*-values adjusted using the BH method.

## Supplementary Material

msag252_Supplementary_Data

## Data Availability

All the sequencing data have been deposited to the NCBI Sequence Read Archive (SRA) under GenBank Bioproject PRJNA1404350. The codes are available at GitHub (https://github.com/ZhaoruiZhou/Intron_Evolution_in_Ciliates).
