# Supplementary material for: Contrasting modes of intron evolution in an evolutionarily distinct eukaryotic lineage revealed by comparative genomics of ciliated protists
Source: Mol Biol Evol. 2026 Sep 30;43(10):msag252. doi: 10.1093/molbev/msag252 (PMC13685798; doi:10.1093/molbev/msag252)
Supplement: Supplementary file 1 [file msag252_supplementary_data.zip › Supplementary.pdf]

Running title: Intron evolution in ciliates

Contrasting modes of intron evolution in an evolutionarily distinct eukaryotic lineage revealed by comparative genomics of ciliated protists

Zhaorui Zhou [1]†, Ruitao Gong [1]†, Yong Chi [2], Yurui Wang [3], Zhiqiang Ye [4], Ying Yan [1]*

[1] *Key Laboratory of Evolution & Marine Biodiversity (Ministry of Education), and Institute of Evolution & Marine Biodiversity, Ocean University of China, Qingdao 266003, China*

[2] *Marine College, Shandong University, Weihai 264209, China*

[3] *Laboratory of Biodiversity and Evolution of Protozoa in Wetland, College of Life Sciences, Shaanxi Normal University, Xi'an 710119, China*

[4] *Key Laboratory of Pesticide & Chemical Biology of Ministry of Education, School of Life Sciences, Central China Normal University, Wuhan 430079, China*

† These authors contributed equally to this work.

* Correspondence: Ying Yan, E-mail: yanying@ouc.edu.cn;

Supplementary Figures

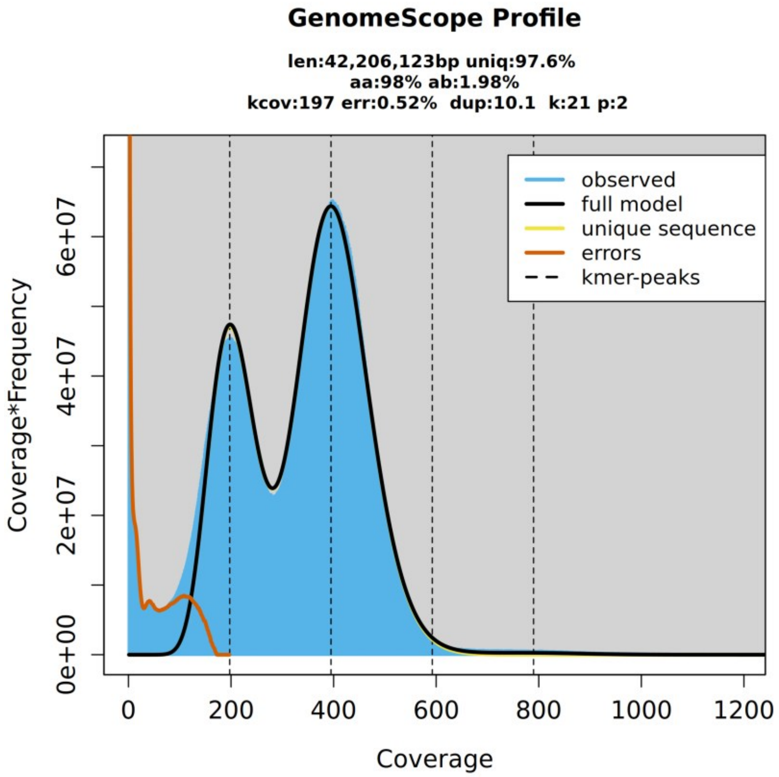

Figure S1. K-mer based genome size estimation of the *B. undulans.* The k-mer spectrum suggested that the genome size is approximately 42.2 Mb.

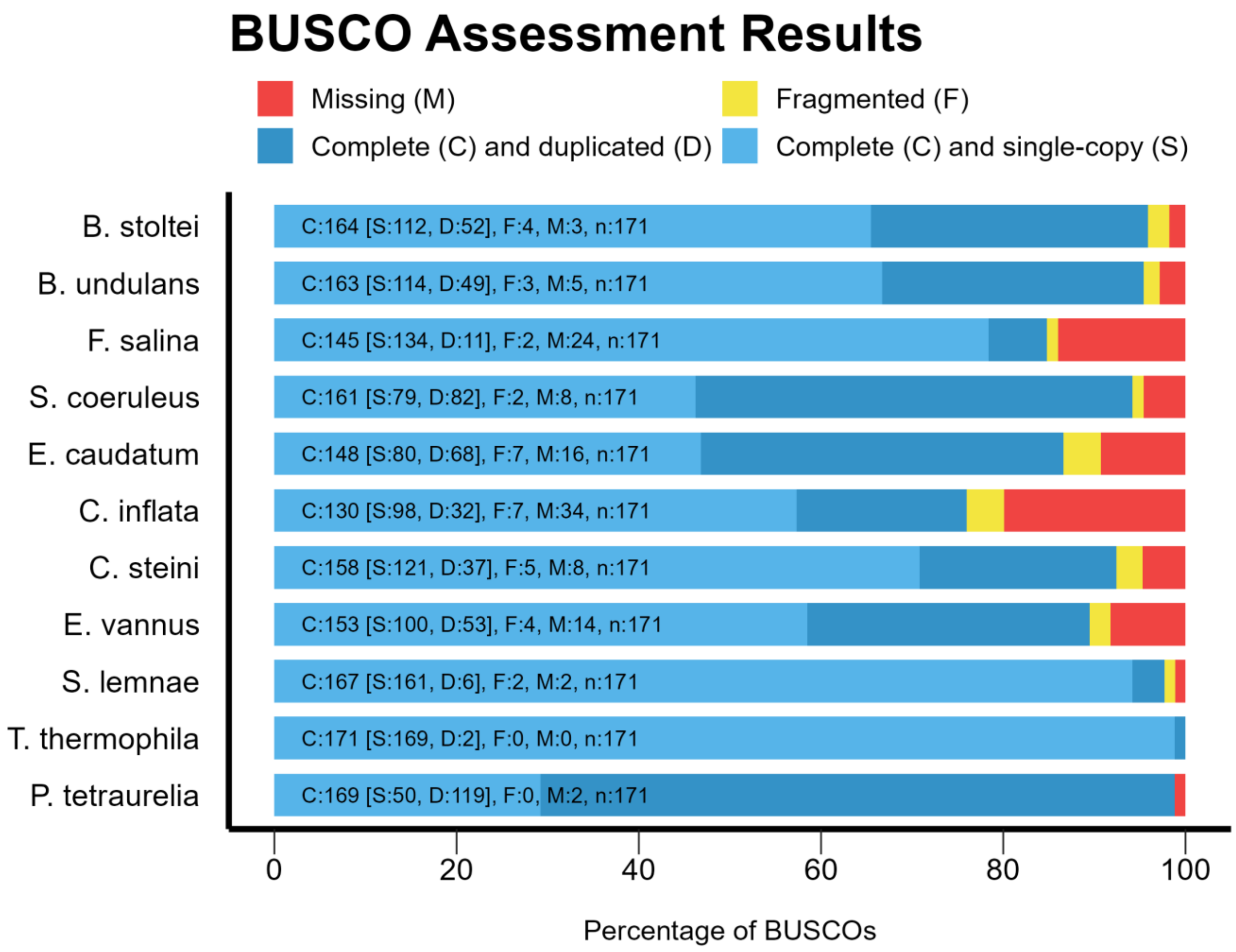

Figure S2. **BUSCO assessment results (protein mode) for representative ciliate species.**

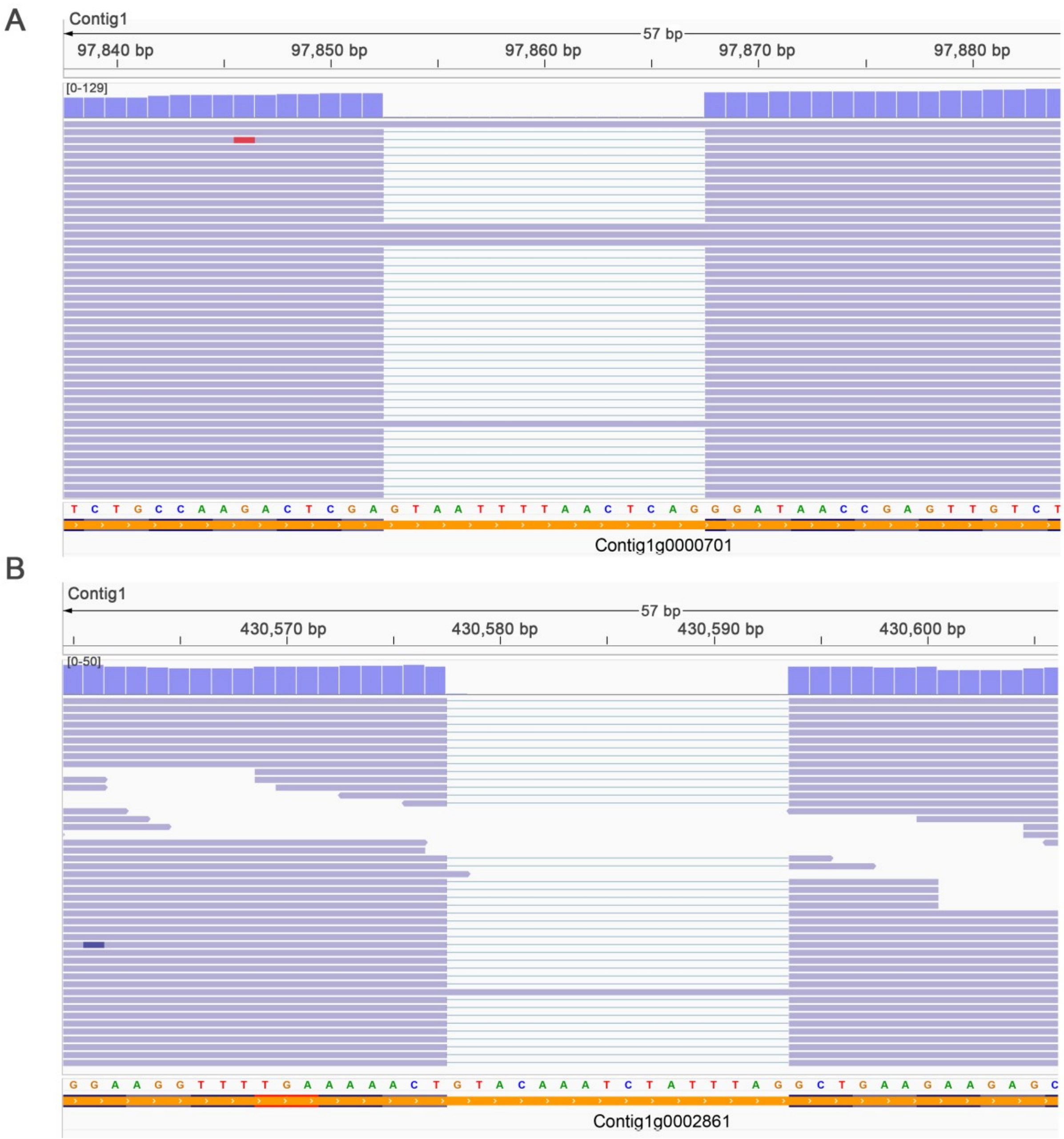

Figure S3. **Visualization of RNA-seq reads alignments supporting short introns in *Blepharisma undulans*.** (A) IGV view of RNA-seq reads aligned to a representative 15 bp intron locus. (B) IGV view of RNA-seq reads aligned to a representative 16 bp intron locus.

=--

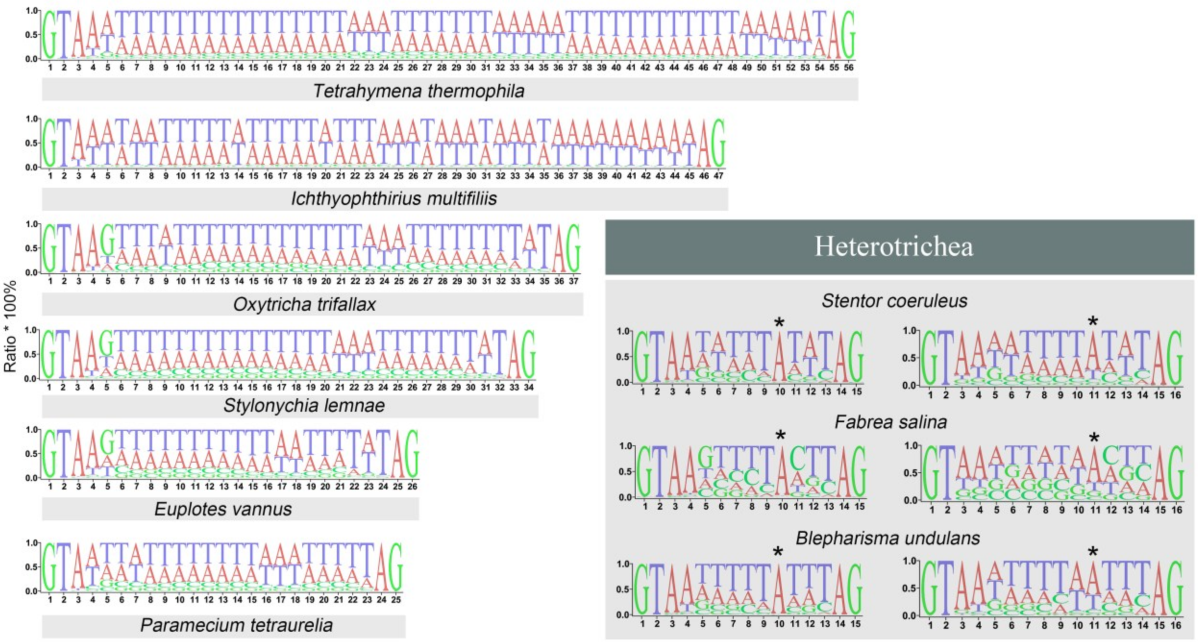

Figure S4. Sequence motif enrichment display of representative intron lengths across multiple ciliates.

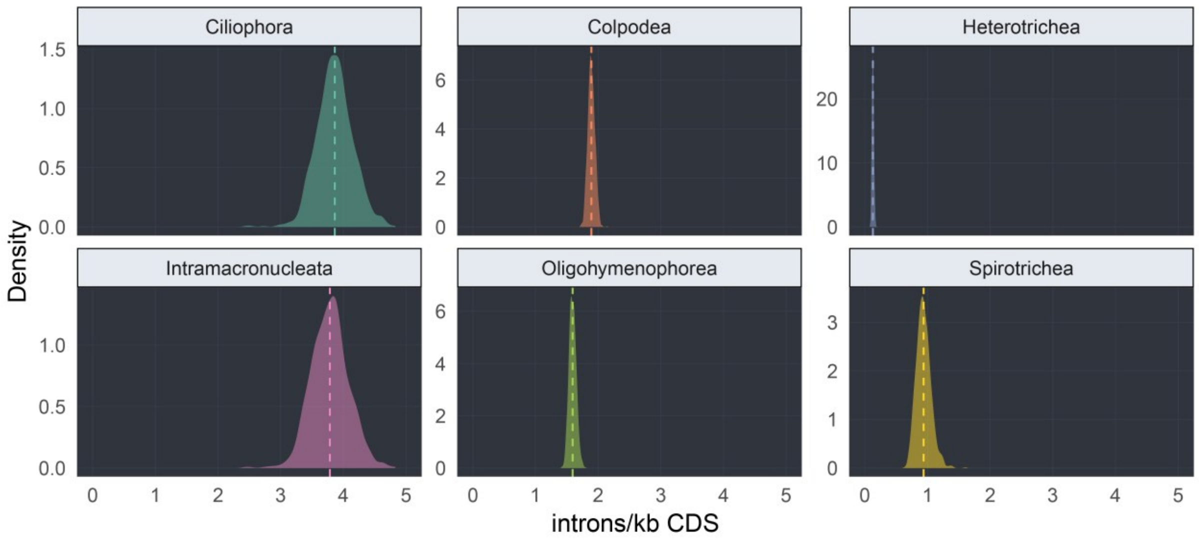

Figure S5. Ancestral intron densities. The ancestral intron densities of the phylum Ciliophora were reconstructed using a Monte Carlo approximation based on 1000 bootstrap distributions, with vertical lines denoting the median values of the 1000 MCMC estimates.

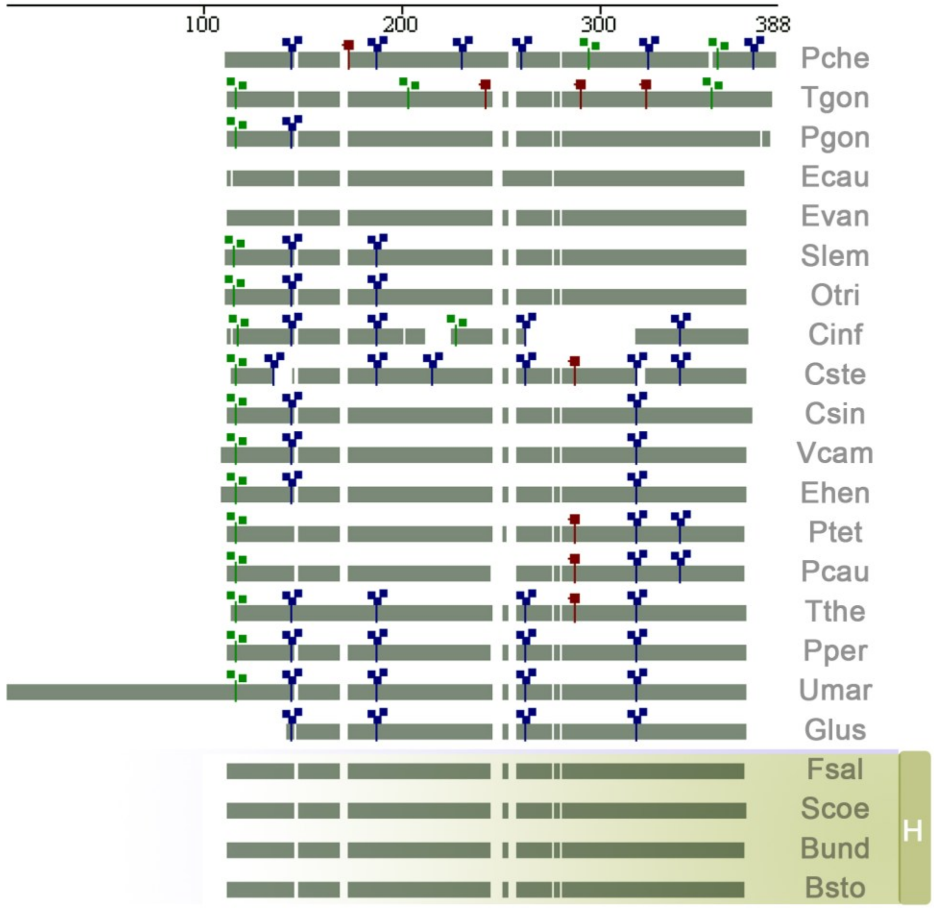

Figure S6. A single orthologous group was selected to illustrate the desert-like distribution pattern of introns in heterotrich ciliates. Introns of different phases are denoted by markers varying in both color and petal count: red for phase 0, green for phase 1, and blue for phase 2.

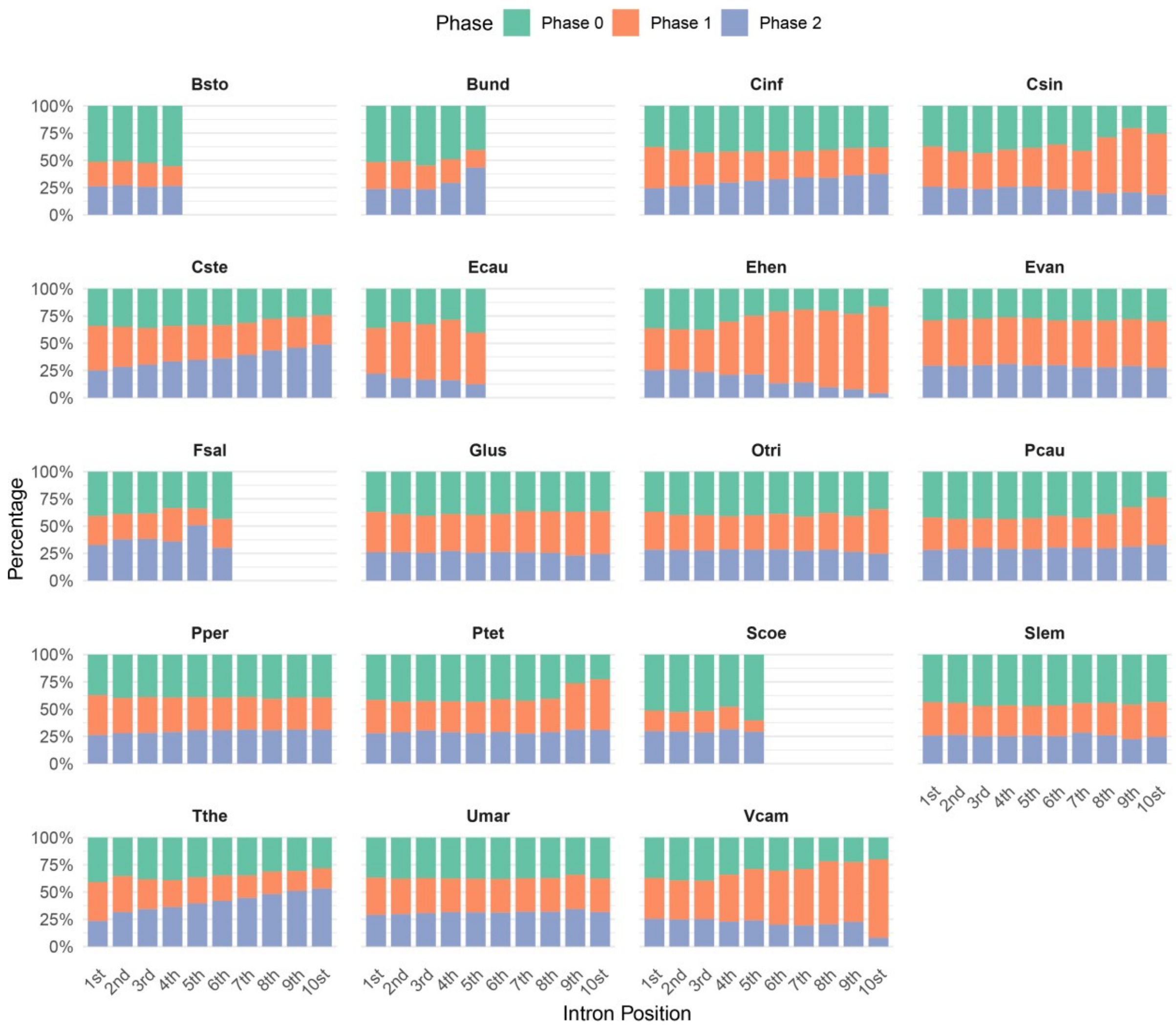

Figure S7. The relationship between intron ordinal position (from 5' to 3' end) and phase distribution in selected ciliates. 1st represents the first intron at the 5' end.

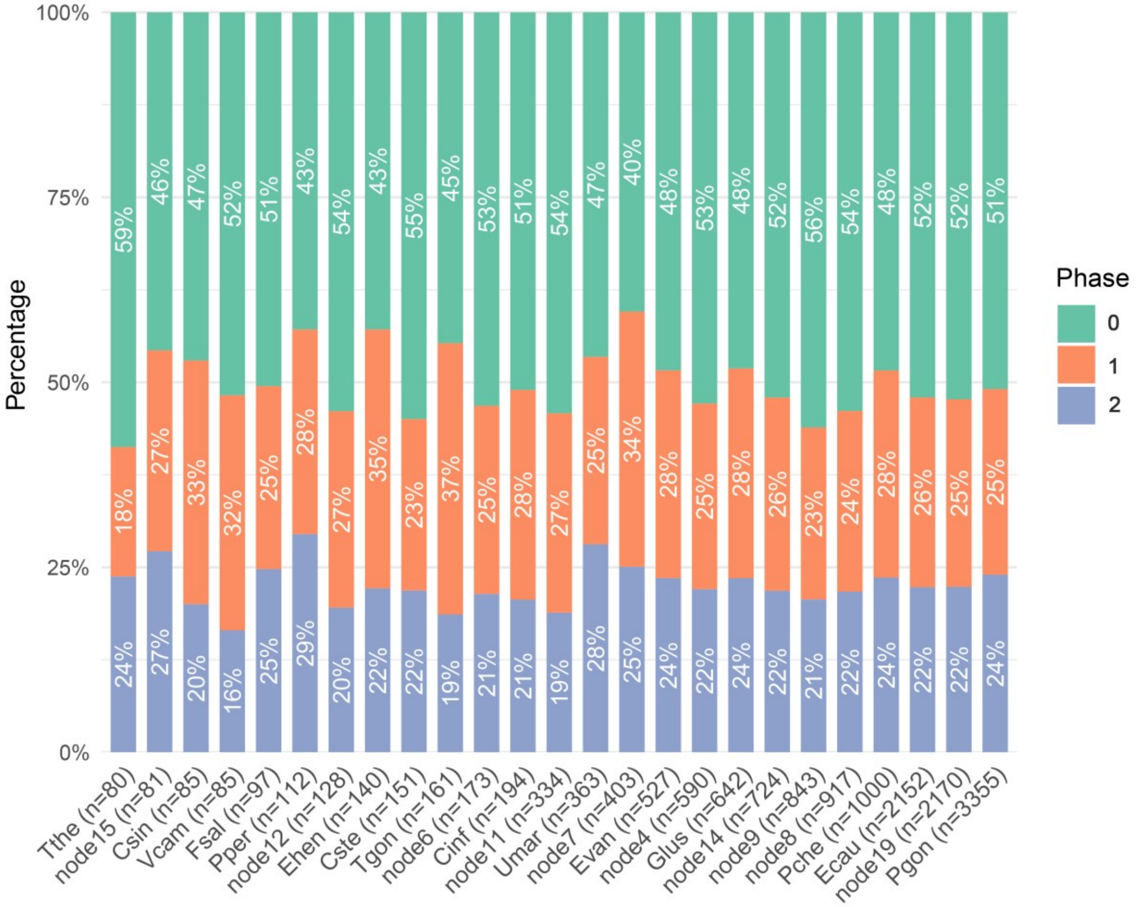

Figure S8. The phase distribution of intron loss at different nodes.

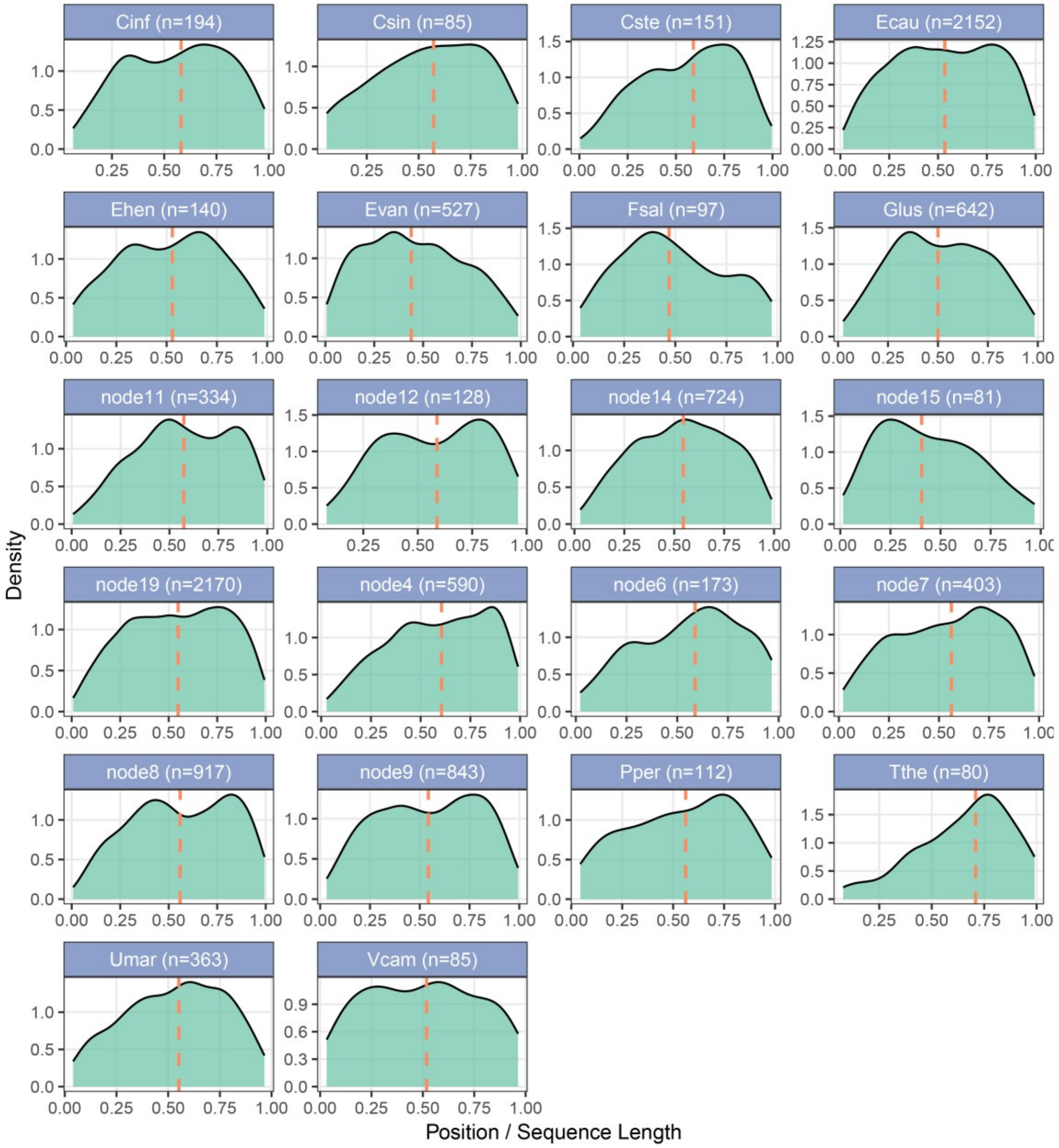

Figure S9. Positional distribution (from 5' to 3' end) bias of lost introns.

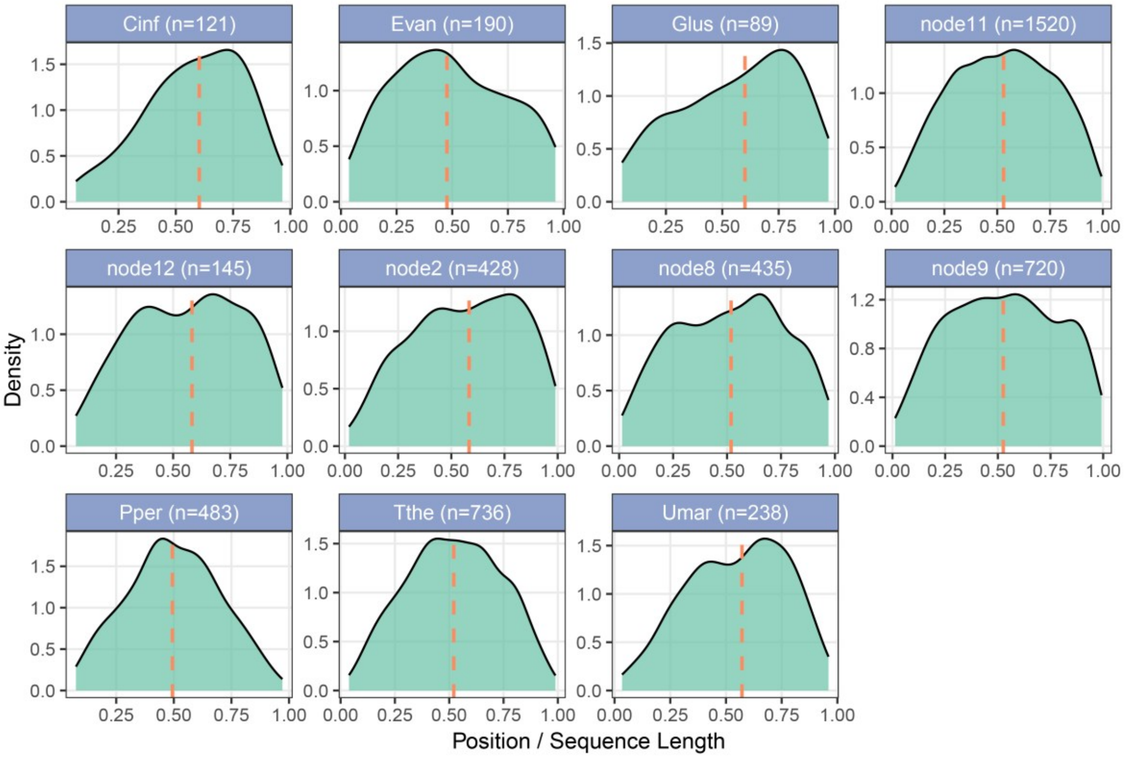

Figure S10. Positional distribution (from 5' to 3' end) bias of gained introns.

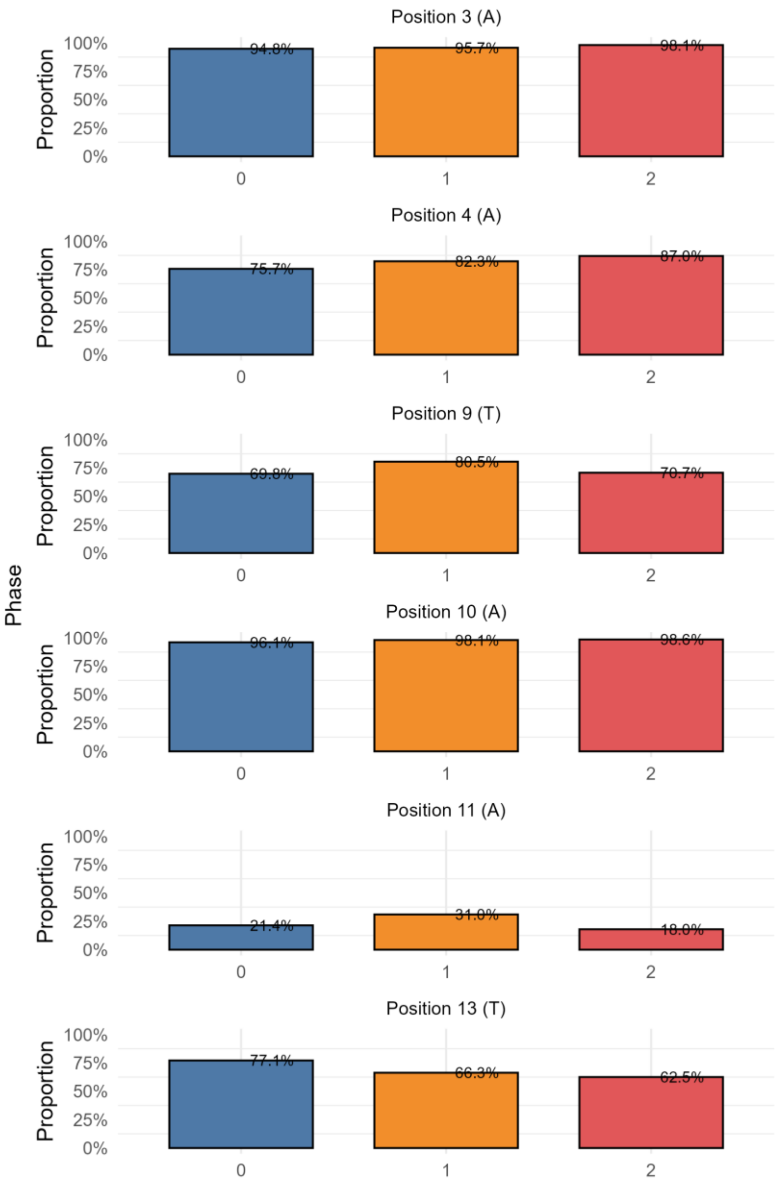

Figure S11. Proportion of specific nucleotides at defined positions within 15 bp introns, categorized by phase.

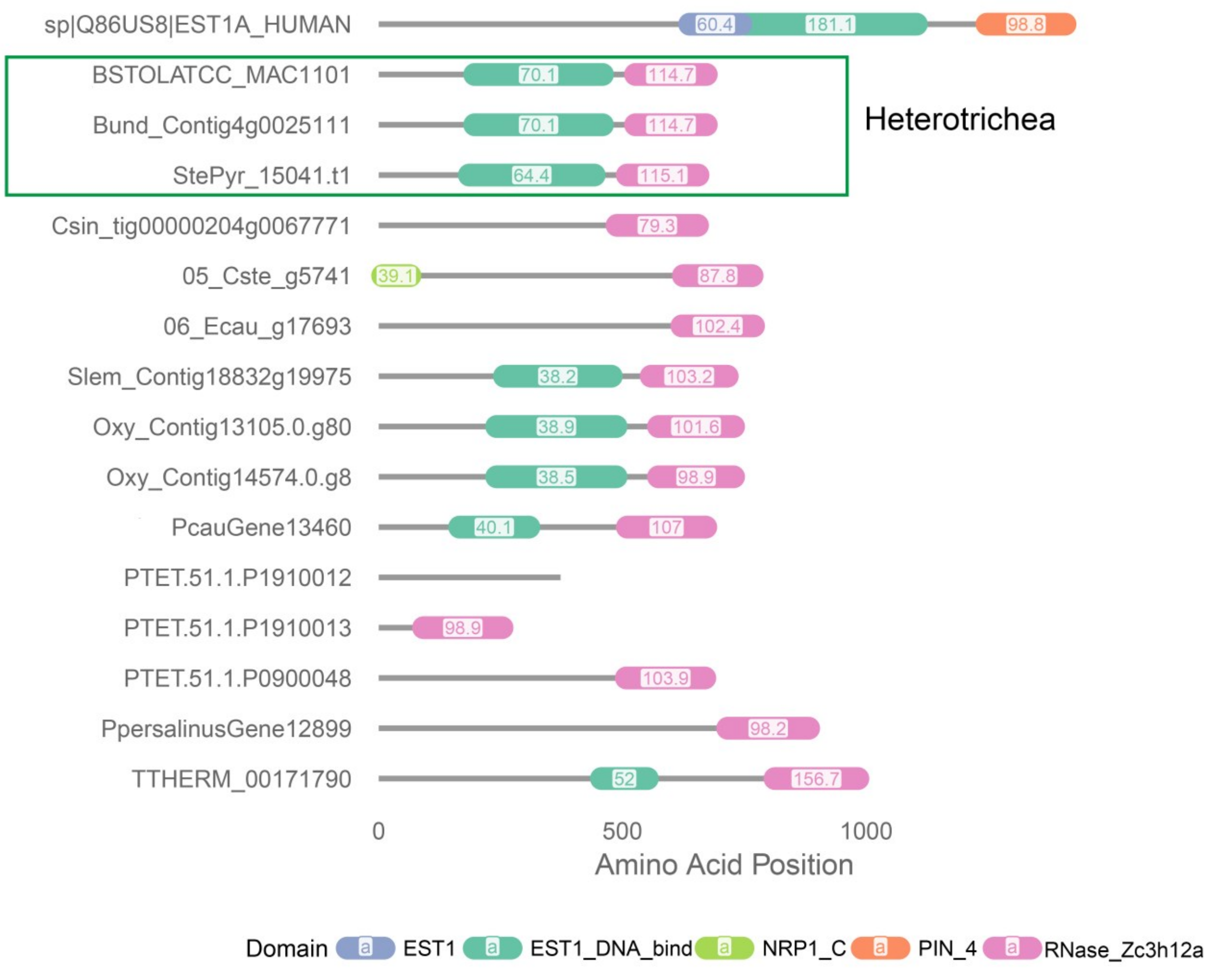

Figure S12. Domain comparison of the SMG6/SMG6L genes between heterotrich ciliates, other ciliates, and human.

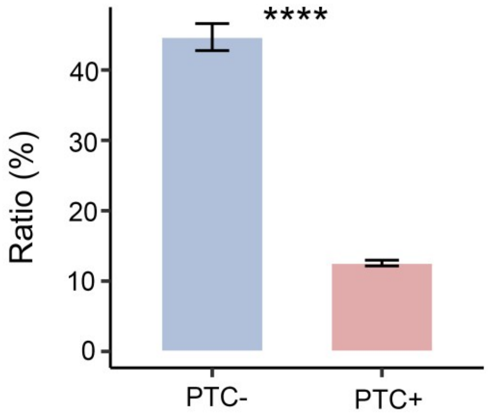

Figure S13. A comparison of the relative retention ratios between 15 bp introns containing PTC and those lacking PTC. **** ($p < 0.0001$, t-test)

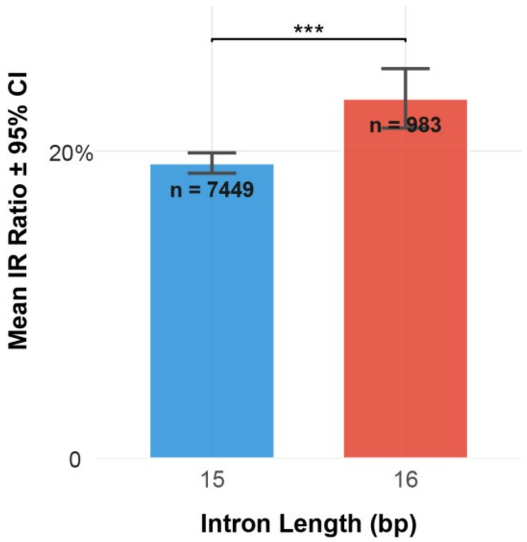

Figure S14. The intron retention ratios comparison between 15 bp and 16 bp introns in *B. undulans*. *** (t-test, $p < 0.001$)

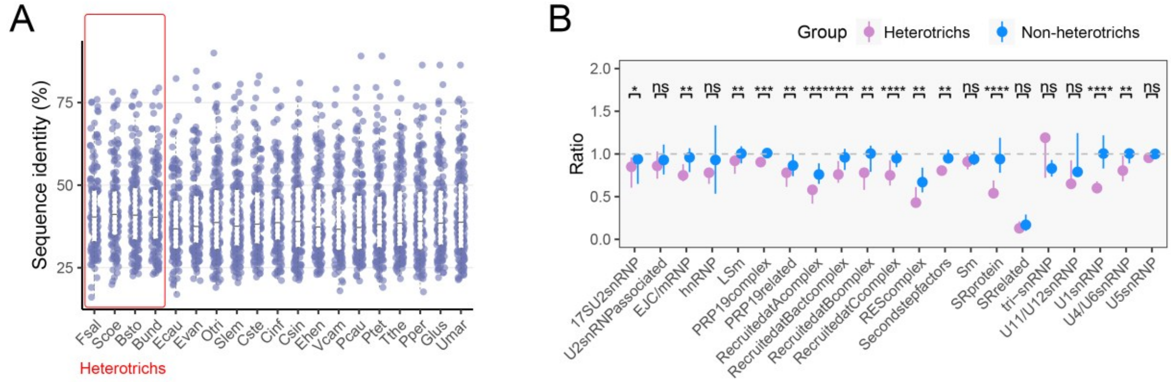

Figure S15. Spliceosome-related protein features in ciliates. (A) BLASTp-based identity statistics of spliceosome-related proteins across ciliates. Each box spans from the 25th to the 75th percentile (first to third quartile), with the central line indicating the median. (B) Comparative analysis of protein length ratios between human spliceosome components and their homologs in heterotrich ciliates and other ciliate lineages. *p* values were determined using the Wilcoxon rank-sum test. Significance levels are indicated as follows: ns = not significant, $p < 0.05$ (*), $p < 0.01$ (**), $p < 0.001$ (***), $p < 0.0001$ (****).

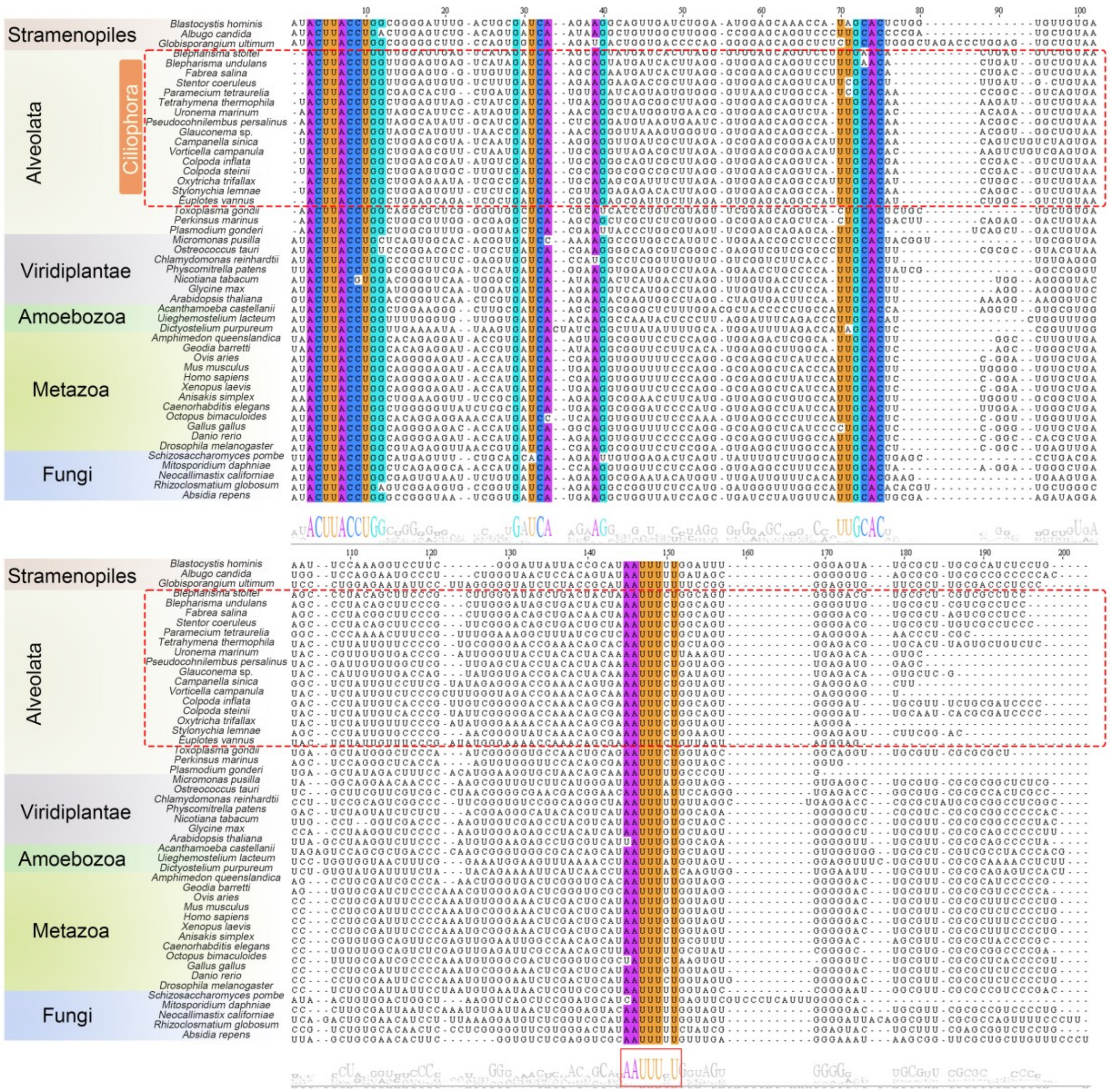

Figure S16. Multiple sequence alignment results of eukaryotic U1 snRNA. Sequences within the dashed box correspond to ciliate sequences. The Sm site conserved motif was outlined with a solid red border.

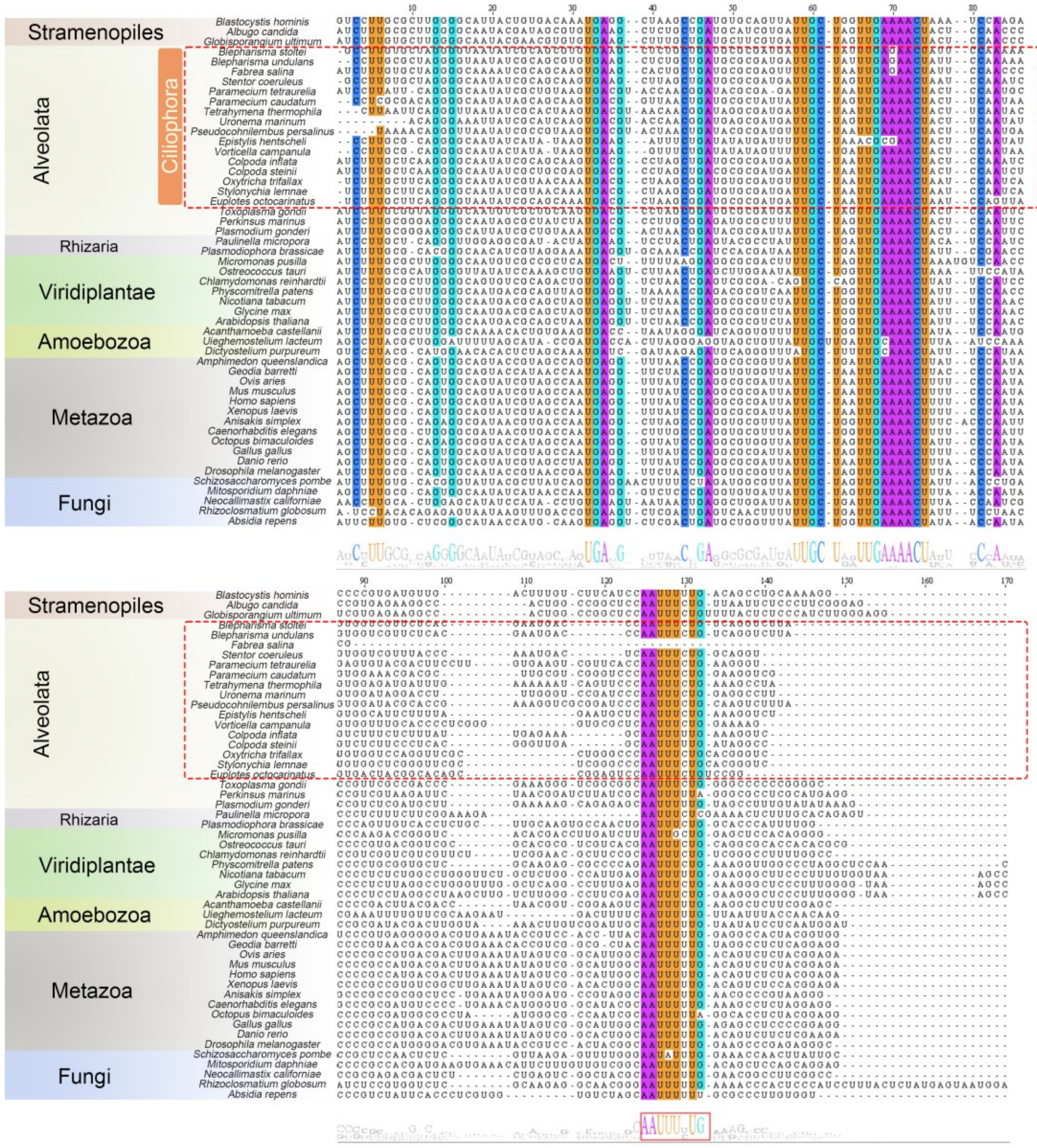

Figure S17. Multiple sequence alignment results of eukaryotic U4 snRNA. Sequences within the dashed box correspond to ciliate sequences. The Sm site conserved motif was outlined with a solid red border.

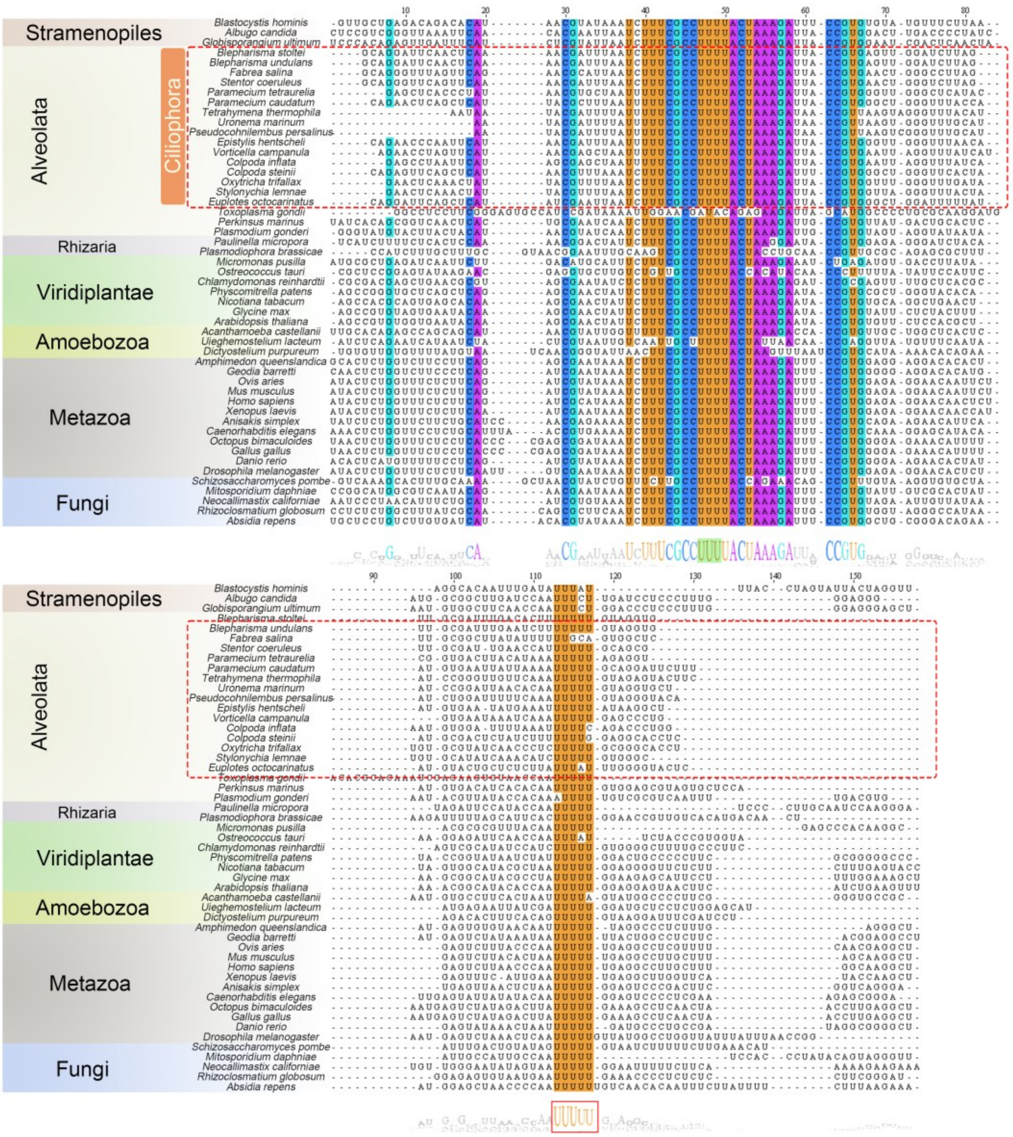

Figure S18. Multiple sequence alignment and secondary structure schematic of eukaryotic U5 snRNA. Sequences within the dashed box correspond to ciliate sequences, with the green background marking the poly-pyrimidine tract binding site for U5 snRNA. The Sm site conserved motif was outlined with a solid red border.

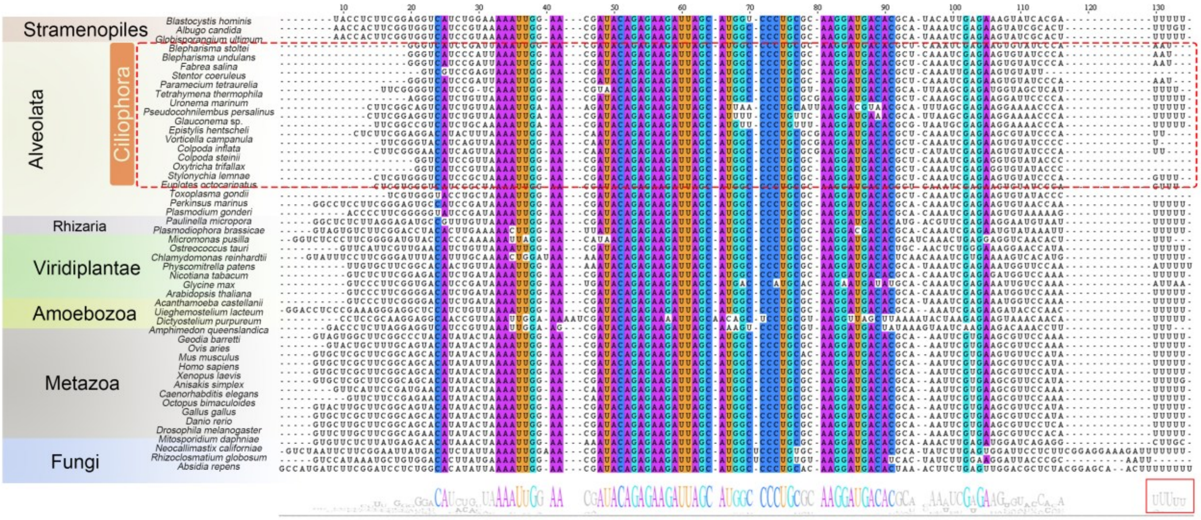

Figure S19. Multiple sequence alignment results of eukaryotic U6 snRNA. Sequences within the dashed box correspond to ciliate sequences. The Sm site conserved motif was outlined with a solid red border.

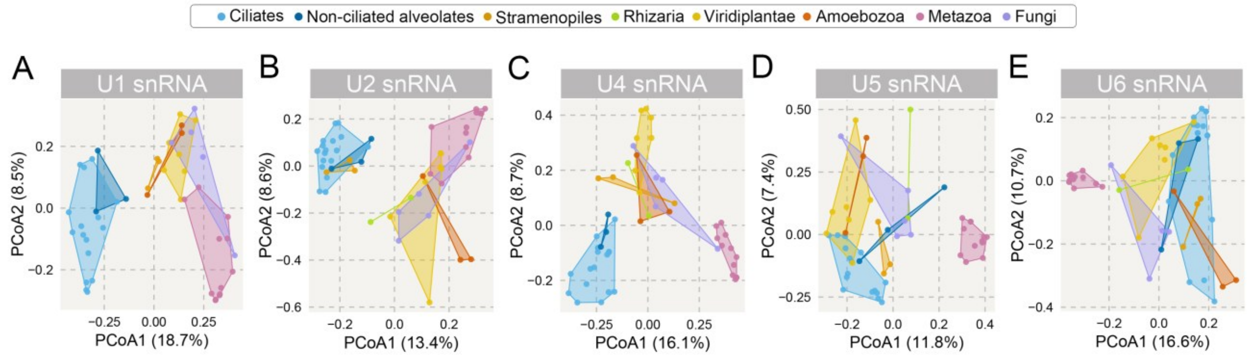

Figure S20. Principal coordinates analysis (PCoA) plots (A-E) based on pairwise sequence similarity of U1, U2, U4, U5, and U6 snRNAs, respectively, across different eukaryotic lineages.

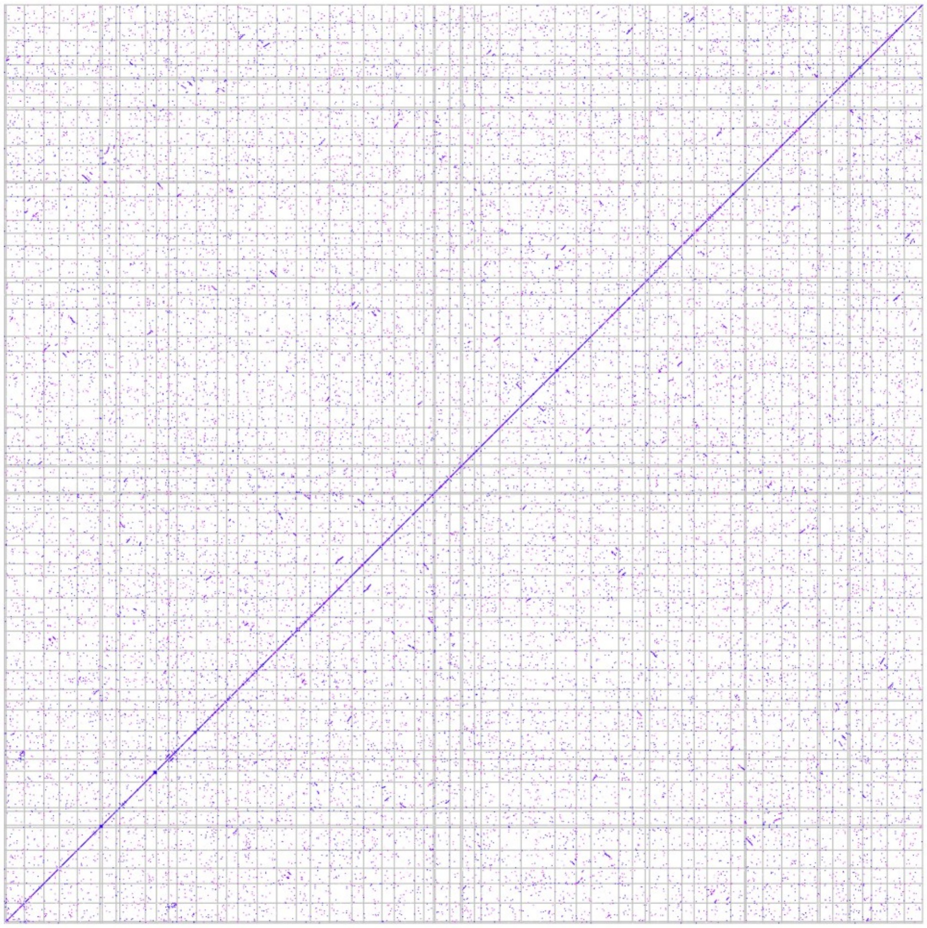

Figure S21. Self-genome dotplot of *Blepharisma stoltei*. Both rows and columns represent individual contigs of the *B. stoltei* genome assembly. Each dot denotes a BLASTp match between two genomic regions.

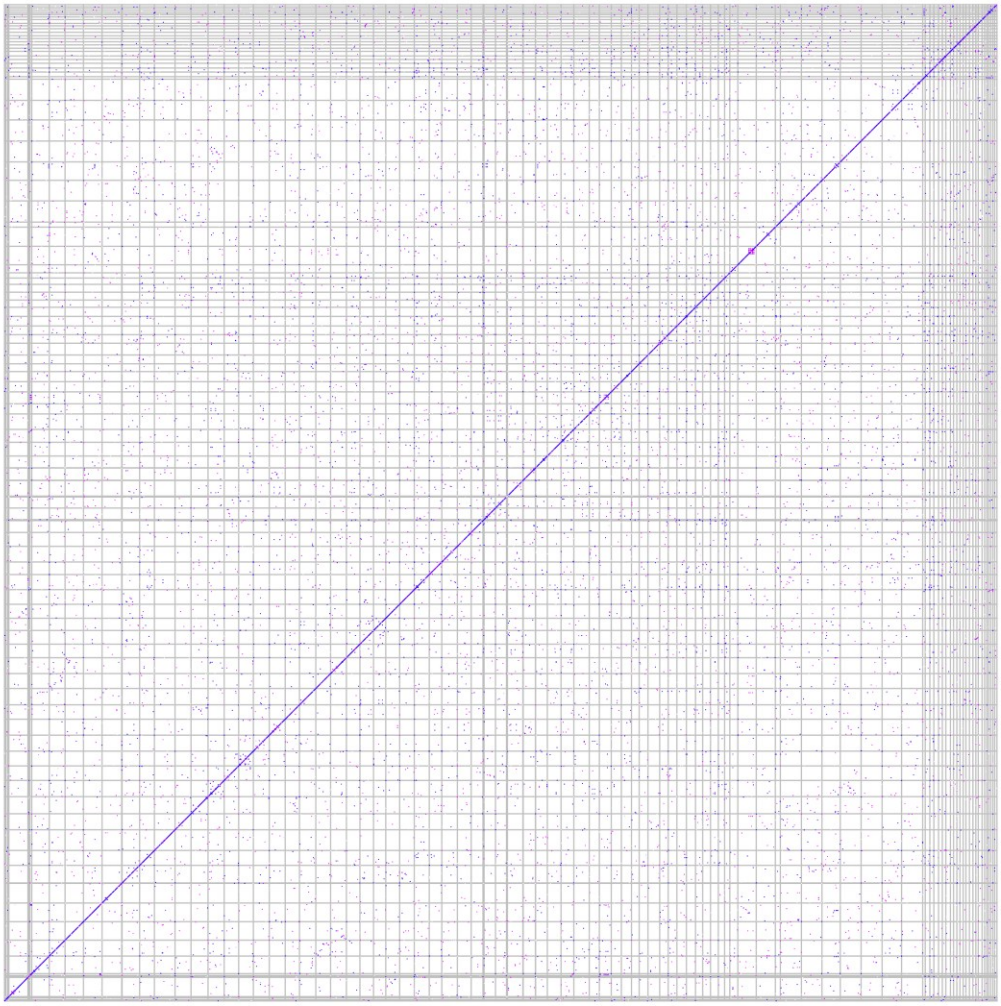

Figure S22. Self-genome dotplot of *Fabrea salina*. Both rows and columns represent individual contigs of the *F. salina* genome assembly. Each dot denotes a BLASTp match between two genomic regions.

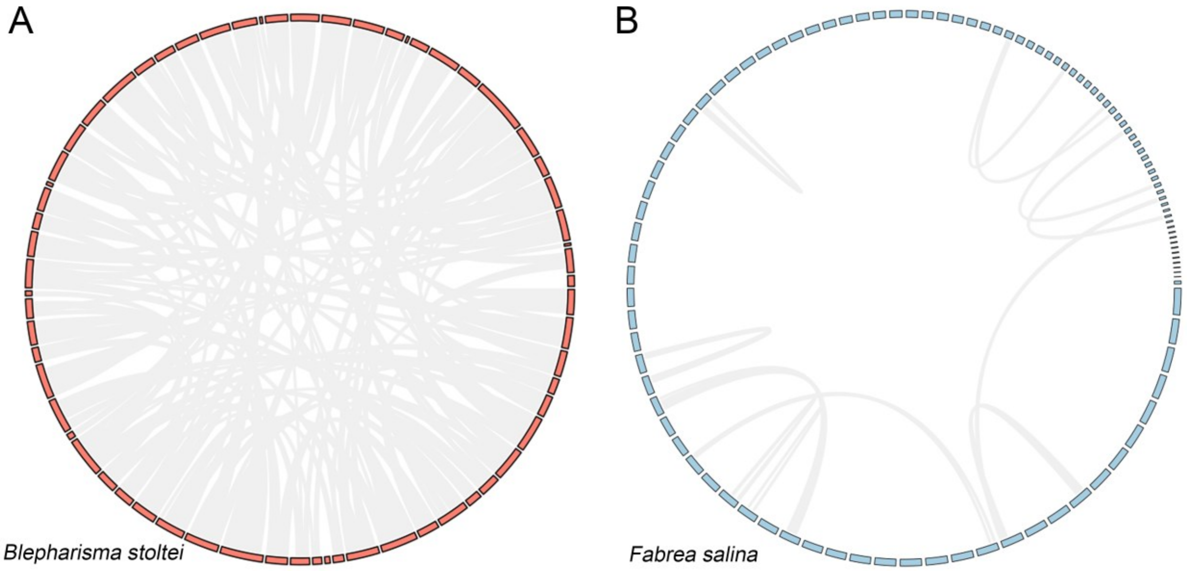

Figure S23. Circos plots of the *B. stoltei* (A) and *F. salina* (B) genomes. Each track represents an individual contig, with contigs arranged around the circle. Gray ribbons in the inner circle connect syntenic gene pairs between different contigs, identified by BLASTp (e-value < 1e−10) with syntenic blocks defined as containing more than five consecutive collinear genes.

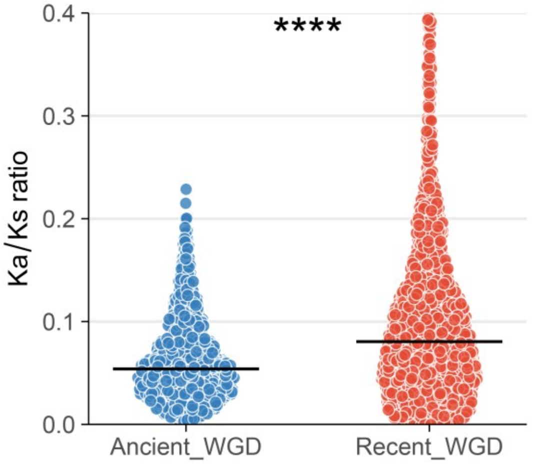

Figure S24. Comparison of *Ka*/*Ks* ratios for gene pairs derived from the two WGD events in *B. undulans*. **** represent a p value < 0.001 (Mann-Whitney U Test).
